# Metabolomics Defines Complex Patterns of Dyslipidaemia in Juvenile-SLE Patients Associated with Inflammation and Potential Cardiovascular Disease Risk

**DOI:** 10.3390/metabo12010003

**Published:** 2021-12-21

**Authors:** George A. Robinson, Junjie Peng, Ines Pineda-Torra, Coziana Ciurtin, Elizabeth C. Jury

**Affiliations:** 1 Centre for Rheumatology Research, Department of Medicine, University College London, London W1CE 6JF, UK; 2 Centre for Adolescent Rheumatology versus Arthritis, Department of Medicine, University College London, London W1CE 6JF, UK; junjie.peng.15@ucl.ac.uk; 3 Centre for Cardiometabolic and Vascular Science, Department of Medicine, University College London, London W1CE 6JF, UK; i.torra@ucl.ac.uk

**Keywords:** juvenile-onset SLE, metabolomics, lipoproteins, lipid rafts, disease activity, cardiovascular disease, atherosclerosis, T-cells, B-cells

## Abstract

Cardiovascular disease (CVD) is a leading cause of mortality in patients with juvenile-onset systemic lupus erythematosus (JSLE) associated with atherosclerosis. The interplay between dyslipidaemia and inflammation—mechanisms that drive atherosclerosis—were investigated retrospectively in adolescent JSLE patients using lipoprotein-based serum metabolomics in patients with active and inactive disease, compared to healthy controls (HCs). Data was analysed using machine learning, logistic regression, and linear regression. Dyslipidaemia in JSLE patients was characterised by lower levels of small atheroprotective high-density lipoprotein subsets compared to HCs. These changes were exacerbated by active disease and additionally associated with significantly higher atherogenic very-low-density lipoproteins (VLDL) compared to patients with low disease activity. Atherogenic lipoprotein subset expression correlated positively with clinical and serological markers of JSLE disease activity/inflammation and was associated with disturbed liver function, and elevated expression of T-cell and B-cell lipid rafts (cell signalling platforms mediating immune cell activation). Finally, exposing VLDL/LDL from patients with active disease to HC lymphocytes induced a significant increase in lymphocyte lipid raft activation compared to VLDL/LDL from inactive patients. Thus, metabolomic analysis identified complex patterns of atherogenic dyslipidaemia in JSLE patients associated with inflammation. This could inform lipid-targeted therapies in JSLE to improve cardiovascular outcomes.

## 1. Introduction

Systemic lupus erythematosus (SLE) is a complex autoimmune disorder characterized by loss of immune cell regulation and the presence of autoantibodies against multiple nuclear and non-nuclear antigens, resulting in chronic inflammation and multiple organ damage. Between 15–20% of all patients with SLE have juvenile-onset disease (JSLE) [1] which is characterized by a more aggressive disease phenotype [2]. Cardiovascular disease (CVD) as a serious long-term complication of SLE and JSLE [3] and is mediated by atherosclerosis, a chronic inflammatory disease of the large arteries, characterised by defects in lipid homeostasis (dyslipidaemia) [4]. Dyslipdaemia refers to an imbalance of systemic lipid transport in the blood, largely mediated by lipoproteins, including atherogenic very low, intermediate, and low density lipoproteins (VLDL, IDL, and LDL), as well as atheroprotective high density lipoproteins (HDL), which conventionally transport lipids (such as cholesterol) to and from atherosclerotic plaques, respectively.

Patients with JSLE have an increased risk of mortality due to CVD [5,6]. This indicates that the atherosclerotic process which is known to begin in childhood or adolescence and is accelerated in JSLE patients due to the prolonged exposure to inflammation [7,8]. Dyslipidaemia is an established risk factor for CVD, but lipids also have potent effects on the immune system [9]. Multiple lipid defects have been described in JSLE/SLE, including dyslipidaemia comprising elevated levels of triglycerides and LDL, depressed levels of HDL and apolipoprotein-A1 (Apo-A1), and an increased abundance of dysfunctional proinflammatory HDL, lacking the antioxidant capacity of conventionally cardioprotective HDL [6,10,11]. Dyslipidaemia in JSLE patients has a complex etiology believed to be dependent on several factors including cytokine levels, specific autoantibodies, dietary intake, genetic factors, hormones, and physical activity.

Lipid rafts are glycosphingolipid (GSL) and cholesterol rich areas of the plasma membrane where signalling molecules accumulate at high density, representing an interface between systemic lipid metabolism and cellular function. Modulation of lipid rafts is associated with both immune cell function and atherogenesis [9,12]. In T-cells from healthy donors, ligation of the T-cell receptor induces rapid lipid raft clustering and amplification of cell signalling and T-cell activation [13,14]. Pre-clustering of lipid rafts is evident in freshly isolated SLE T-cells and may favour pre-activation of signalling proteins contained within lipid rafts and accelerate downstream signalling [13,15]. This raises the possibility that drugs inhibiting lipid biosynthesis could be beneficial in patients with SLE through the dampening of T-cell receptor signalling [16,17]. Very little information is available to assess whether defects in lipid rafts and/or lipid biosynthesis are present in the immune cells of patients with JSLE. Changes in serum cholesterol transport via LDL and HDL can also influence lipid rafts and immune cell function by modifying immune cell activation signals and could result in a more inflammatory and pro-atherogenic local response [4,18,19]. Interplay between traditional cardiovascular risk factors (such as dyslipidaemia) and risk factors associated with continuing (J)SLE disease, including the altered composition of lipid rafts on immune cells, could create the perfect environment for development of atherosclerosis [4].

Here, the in-depth serum lipoprotein taxonomy of JSLE patients with both clinically active and inactive disease (a degree of serological activity in the context of clinically quiescent disease was permitted) compared to age matched healthy controls (HCs) was defined using metabolomic analysis. The relationship between lipoproteins and immune cell lipids was also explored. High disease activity in JSLE was associated with increased VLDL, IDL, and LDL and decreased HDL. Elevated atherogenic lipoproteins correlated positively with disease activity measures and T- and B-cell lipid raft expression and activation. Therefore, this work suggests a relationship between lipoproteins in circulation and immune cell function.

## 2. Results

### 2.1. Dyslipidaemia in JSLE Patients Is Associated with Reduced Atheroprotective Small HDL Lipoproteins

**Figure 1:**
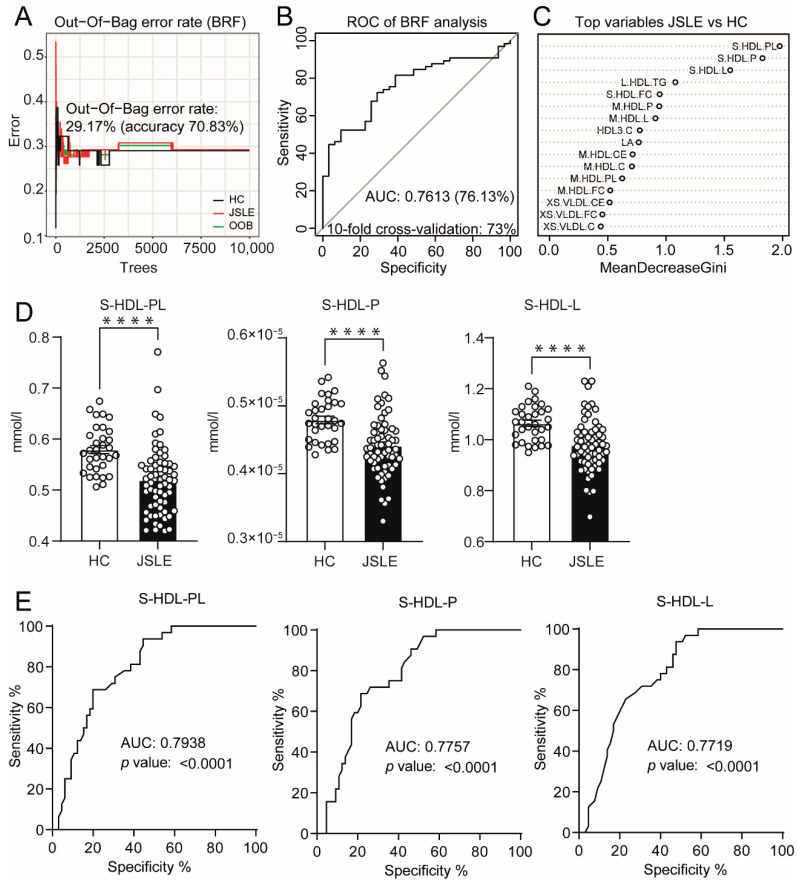
Dyslipidaemia in JSLE is associated with reduced atheroprotective small HDL lipoproteins. (**A**–**C**) Comparison of 130 different lipid metabolites (Supplementary Table S1) in healthy controls (HCs, *n* = 32) versus juvenile-onset SLE (JSLE, *n* = 67) using the balanced random forest (BRF) machine learning model as described in the methods. Demographic variables including gender, age, and race were adjusted in the model. (**A**) The out of bag (OOB) error rate was 29.17%. (**B**) Reciever operator characteristic (ROC) curve analysis and 10-fold cross validation was used to validate the model providing an area under the curve (AUC) of 0.7613 and an accuracy of 73%. (**C**) The top 10 variables contributing to the BRF model are shown. The mean decrease in Gini measures the importance of each variable to the model: a higher score indicates a higher importance of the variable. (**D**,**E**) Individual scatter plots (**D**) and ROC plots (with AUC) (**E**) of the top 3 metabolites from the BRF predictive model comparing HCs (*n* = 32) to JSLE patients (*n* = 65). Unpaired *t* test. Mean. SEM. **** = *p* < 0.0001. Abbreviations: VLDL, very low-density lipoprotein; HDL, high density lipoprotein; XS, very small; S, small; M, medium; L, large; C, cholesterol; CE, cholesterol ester; FA, fatty acid; FC, free cholesterol; L, total lipids; P, particles; PL, phospholipids; TG, triglycerides; LA, linoleic acid. For patients the SLEDAI score was calculated, a score of 4 or higher represents active disease [20]. Other common clinical measures of disease are shown as well as treatments. Rituximab treatment was avoided in the cohort. Fisher’s exact test or one-way ANOVA* was used. Abbreviations: BMI: Body Mass Index, ENA: Extractable Nuclear Antigens, NR: Normal ranges, SLEDAI: Systemic Lupus Erythematosus Disease Activity Index, dsDNA: Anti-double-stranded-DNA antibodies, C3: Complement component 3, LC: Lymphocyte count, HDL-C: High density lipoprotein cholesterol, LDL-C: Low density lipoprotein cholesterol.

Using an in-depth nuclear magnetic resonance lipid metabolomic platform, a detailed retrospective analysis of the serum lipid profile of adolescent patients with JSLE was performed compared to age-matched HCs (Table 1 for cohort characteristics, Supplementary Table S1 for list of metabolic biomarkers). Using a balanced random forest (BRF) machine learning approach, 130 lipid metabolites were analysed—adjusting for age, sex, and race (Figure 1A–C). The BRF model distinguished JSLE patients from HCs with a 76% prediction accuracy (73% by 10-fold cross-validation) (Figure 1B), supporting a distinctive dyslipidaemic profile in adolescent patients with JSLE. The variables contributing most to the model were small (S)-HDL subsets, including total lipid (L) and phospholipid (PL) content, and S-HDL particle concentration (P) followed by medium HDL subsets, linoleic acid and very small (XS)-VLDL subsets (Figure 1C). These results were confirmed when the expression of the top three individual metabolites differentiating patients from HCs (mean decrease in Gini>1.5; S-HDL-PL, S-HDL-P, and S-HDL-L) were assessed by conventional statistical analysis (Figure 1D, Supplementary Figure S1A for other metabolites). Notably, receiver operator characteristic (ROC) analysis of these metabolites showed an AUC of 0.796, 0.779, and 0.774 respectively (Figure 1E), suggesting that atherogenic dyslipidaemia in JSLE may be compartmentalised to circulating S-HDL lipid levels, as opposed to the entire lipidome as shown by the BRF model. Importantly, these HDL metabolites were not significantly associated with the levels of circulating anti-ApoA1 antibodies (Supplementary Table S2) and there was no significant difference in ApoA1 expression or the HDL-C:ApoA1 ratio between patients with JSLE and HCs (Supplementary Figure S1B), suggesting that global and HDL-associated ApoA1 expression were not altered in JSLE.

### 2.2. Atherogenic Lipoproteins Are Associated with Increased Disease Activity in JSLE

**Figure 2:**
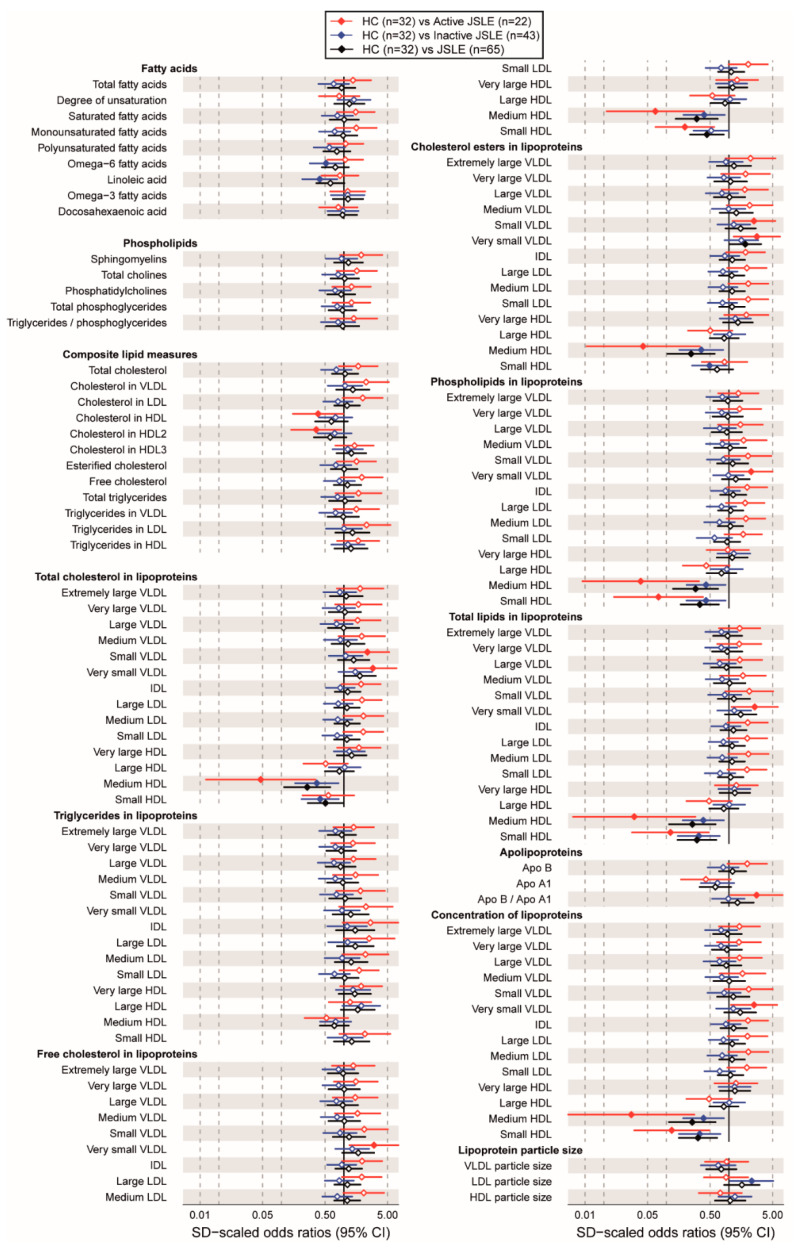
Atherogenic lipoproteins are associated with increased disease activity in JSLE. (**A**) Forest plot showing odds ratios (ORs) and 95% confidence intervals (CIs) of serum lipid metabolites between all JSLE patients (*n* = 65), and those with clinically active (*n* = 22) and inactive (*n* = 43) disease compared to healthy controls (HCs, *n* = 32) by logistic regression analysis adjusted for age, sex, and race. The concentration of total lipids (mmol/L), apolipoproteins (g/L), and lipoprotein measurements including particle size (nm), concentration, and lipid content (mmol/L) are displayed. Statistically significant differences denoted by solid black diamond; non-statistically significant differences denoted by open diamond. Abbreviations: Apo, apolipoprotein; VLDL, very low-density lipoprotein; IDL, intermediate density lipoprotein; LDL, low density lipoprotein; HDL, high density lipoprotein. HC vs. all JSLE, black line; HC vs. active JSLE, red line; HC vs. inactive JSLE, blue line.

To support the results of the BRF model, a univariate logistic regression was performed (adjusted for cohort age, sex, and race) comparing the total JSLE cohort, patients with clinically active disease, and patients with inactive disease (assessed using SLE disease activity index, SLEDAI) to HCs (Figure 2, Supplementary Table S3). S-HDL and M-HDL particle concentration and lipid content (phospholipids, cholesterol esters, free cholesterol, total cholesterol, and total lipids) were significantly reduced in all JSLE patients compared to HCs, as identified in the BRF model. These differences were exacerbated in patients with more active disease compared to HCs, despite seeing no significant differences in clinical lipid levels between active and inactive JSLE patients (Table 1). Interestingly, XS-VLDL particle concentration and lipid content (phospholipids, cholesterol esters, free cholesterol, total cholesterol, and total lipids), S-VLDL lipid content (cholesterol esters and total cholesterol), and ApoB:ApoA1 were significantly increased only in patients with more active disease, suggesting atherogenic lipoprotein subsets were associated with disease flare.

**Figure 3:**
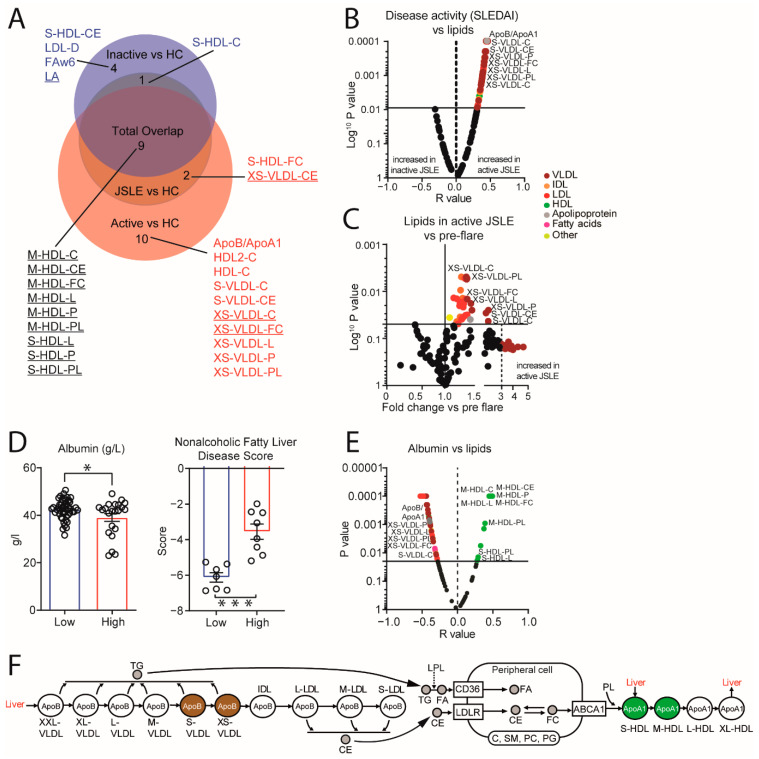
Atherogenic lipoproteins correlate positively with markers of JSLE disease activity and are associated with markers of liver damage. (**A**) Venn diagram (http://www.biovenn.nl/, Accessed on 20 June 2020) displaying the proportional overlap of statistically significantly altered metabolites from (**A**) that overlap between the different group comparisons. Metabolites that are both top variables in the BRF model and are significantly altered by logistic regression analysis are underlined. Abbreviations: Apo, apolipoprotein; VLDL, very low-density lipoprotein; LDL, low density lipoprotein; HDL, high density lipoprotein; C, cholesterol; CE, cholesterol ester; D, diameter; FA, fatty acid; FC, free cholesterol; L, total lipids; P, particles; PL, phospholipids; FAw6, omega-6 fatty acid; LA, linoleic acid. Volcano plot showing (**B**) the correlation (r-value, Pearson’s) of systemic lupus erythematosus disease activity index (SLEDAI) with serum lipid metabolomic measures in JSLE patients (*n* = 65) and (**C**) the fold change of serum lipids in JSLE patients experiencing a clinical flare (previously inactive patients with an increase in SLEDAI by 4 points or more [21], mean increase of 7.2 points)) compared to matched patients pre-flare (*n* = 5). Log10 *p* values are displayed. Horizontal line represents adjusted *p* value threshold following 10% false discovery rate adjustment for multiple comparisons. Coloured dots represent different metabolomic groups. Important metabolites from the Venn diagram (**B**) are labelled. (**D**) Liver function measurements including albumin and non-alcoholic fatty liver disease score in clinically active (*n* = 22 and 7 respectively) versus clinically inactive disease (+/− serological activity) (*n* = 43 and 8 respectively) JSLE patients. Mean, *t*-test. *** *p* = <0.0001, * *p* < 0.05 (**E**) Volcano plot showing the correlation (r-value, Pearson’s) of albumin with serum lipid metabolomic measures in JSLE patients (*n* = 65). Important metabolites from the Venn diagram (**B**) are labelled. Log10 *p* values are displayed. Horizontal line represents adjusted *p* value threshold following 10% false discovery rate adjustment for multiple comparisons. Coloured dots represent different metabolomic groups as per (B–C). (**F**) Schematic representation of known lipoprotein metabolic processes including peripheral cell lipid influx (via CD36 and LDLR) and efflux (via ABCA1), adapted from Frishberg, A. et al. [26]. Metabolites are highlighted that are significantly altered in all JSLE patients (small/medium-HDL, S/M-HDL) and JSLE patients with clinically active disease (small/very small-VLDL, S/XS-VLDL) compared to HCs as informed and validated by analysis from Figure 1 and Figure 2. Abbreviations: ABCA1, ATP Binding Cassette Transporter A1; LDLR, LDL receptor; SM, sphingomyelin; PG, phosphoglycerides; PC, phosphatidylcholine.

This was supported by a Venn analysis, where S/XS-VLDL subsets and ApoB:ApoA1 were significantly dysregulated in patients with clinically active disease only (Figure 3A). Multiple atherogenic VLDL subsets (including those associated exclusively with active disease, Figure 3A), LDL subsets and ApoB:ApoA1 correlated positively with SLEDAI disease activity score (Figure 3B and Supplementary Table S4). Furthermore, longitudinal comparison of metabolomic profiles in patients pre and post disease flare (previously inactive patients with an increase in SLEDAI by 4 points or more [21], mean increase of 7.2 points), identified up to a 5-fold increase (in the case of some VLDL subsets) in atherogenic VLDL and LDL lipoprotein subsets (Figure 3C, Supplementary Table S5). These findings could have substantial clinical implications, since the total VLDL-C levels in JSLE during a flare increased from 0.44 +/− 0.22 mmol/L (mean +/− SD) pre-flare to 0.87 +/− 0.42 mmol/L, well above the normal ranges identified in a clinical setting by the American Association for Clinical Chemistry (AACC, 0.77 mmol/L) [22]. The relationship between active disease and increased VLDL/LDL subsets was maintained when JSLE patients with active vs. inactive disease were compared directly, accounting for demographic features and treatment (Supplementary Figure S2). Of note, differences in S-HDL and M-HDL subsets were identified in all comparisons which may explain their high importance for JSLE prediction in the BRF model. Finally, linoleic acid and total omega-6 fatty acids and LDL diameter were decreased in patients with low active disease only (Figure 3A).

Lipoprotein biosynthesis occurs in the liver and changes in liver function during inflammation are associated with altered circulating lipoprotein levels [23,24]. Patients with clinically active disease had decreased clinical measures of albumin (a decrease indicates reduced liver function) and increased non-alcoholic fatty liver disease (NAFLD) fibrosis scores (a non-invasive score that identifies liver fibrosis [25]) (Figure 3D), suggesting altered liver function. Albumin levels correlated negatively with atherogenic lipoprotein measures (VLDL/LDL/ApoB:ApoA1) and positively with HDL (Figure 3E). Therefore, changes in liver function associated with increased inflammation and lower albumin levels in active JSLE disease likely contribute to elevated VLDL/LDL and reduced HDL levels.

Thus, dyslipidaemia in JSLE is complex, potentially involving multiple mechanisms affecting lipoprotein metabolism (summarised in Figure 3F). How such changes in circulating lipoprotein profiles influence peripheral cellular lipid metabolism in immune cells remains uncertain.

### 2.3. Lipid Raft Signalling Platforms Correlate with Lipoprotein Expression in Active JSLE Patients

**Figure 4:**
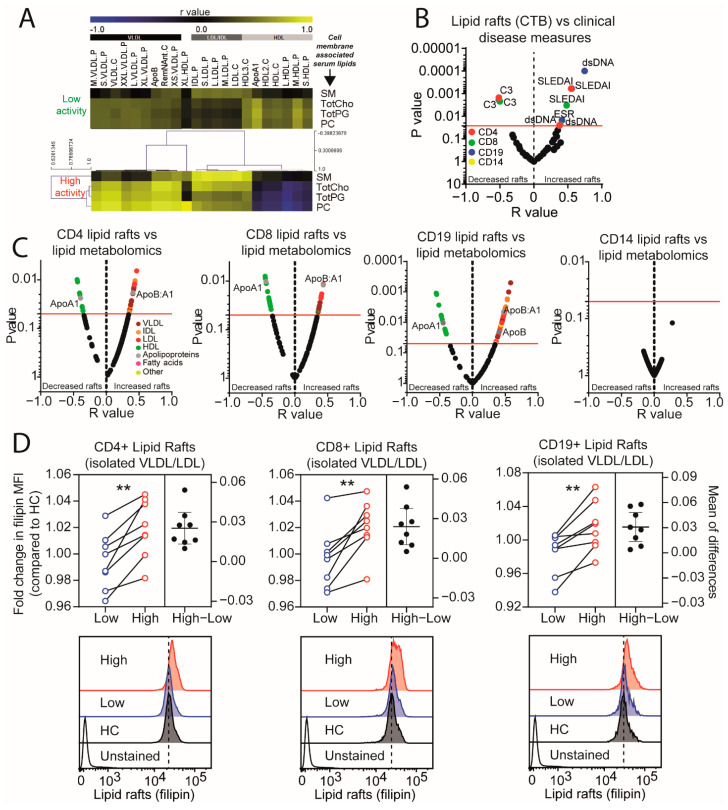
Lymphocyte inflammatory lipid signalling platforms are associated with disease activity and circulating lipoproteins in JSLE. (**A**) Heat map displaying r values (Pearson’s) of the correlation between lipoprotein particles (VLDL, very low-density lipoprotein; IDL, intermediate density lipoprotein; LDL, low density lipoprotein; HDL, high density lipoprotein), including apolipoproteins (ApoB and ApoA1), and serum lipids associated with lipid rafts: SM (sphingomyelin), TotCho (total cholesterol), TotPG (total phosphoglycerides), and PC (phosphatidylcholine and other cholines). (**B**) Volcano plot displaying the correlation (r-value, Pearson’s) of CD4+ and CD8+ T-cell, CD19 B-cell, and CD14 monocyte lipid raft expression (cholera toxin B, CTB; mean fluorescence intensity, MFI, measured ex vivo by flow cytometry) with clinical measures of disease activity (systemic lupus erythematosus disease activity index; SLEDAI, erythrocyte sedimentation rate; ESR, dsDNA; anti-double-stranded DNA antibodies, C3; complement protein 3) in JSLE patients (*n* = 35). Log10 *p* values displayed. (**C**) Volcano plot showing the correlation (r-value, Pearson’s) of CD4+, CD8+ T-cell, CD19 B-cell, and CD14 monocyte lipid raft expression with lipid metabolomic measures in JSLE patients (*n* = 35). Log10 *p* values are displayed. Red line = adjusted *p* value following 10% false discovery rate adjustment for multiple comparisons. Coloured dots represent different metabolomic groups. (**D**) Dot plots displaying the fold change in HC lymphocyte (*n* = 8) lipid rafts (mean fluorescence intensity of filipin, flow cytometry) following in vitro culture of PBMCs with VLDL/LDL isolated from pooled serum from JSLE patients with high (*n* = 9) or low (*n* = 9) disease activity compared to VLDL/LDL from HCs (*n* = 9). PBMCs were cultured for 48hrs in RPMI supplemented with 10% isolated VLDL/LDL from matched volumes of pooled serum (to match physiological levels) for each group. The mean of differences in fold change are displayed on the right axis. ** = *p* < 0.01. Representative histograms of filipin expression are displayed for each group or unstained control. Dashed lines represent the histogram peak for HCs.

We hypothesised that alterations in lipoprotein metabolism and circulating lipids could influence peripheral immune cell lipid uptake and efflux [27] (Figure 3F). Immune cell dysfunction and inflammation in SLE are associated with increased accumulation of lipid raft signalling platforms [13,15,16,17]. Notably, in clinically active disease, circulating atheroprotective HDL subsets had a negative correlation, and atherogenic lipoprotein subsets (LDL/VLDL) had a positive correlation, with circulating lipids typically enriched in lipid raft microdomains (sphingomyelin, cholesterol, phosphoglycerides, phosphatidylcholine, and other cholines, Figure 3F and Figure 4A); conversely, these correlations were reversed or decreased respectively in patients with clinically inactive disease. CD4+ T-cell, CD8+ T-cell, and B-cell lipid raft expression (assessed using the surrogate lipid raft marker cholera-toxin B [16,28]) were increased in patients with clinically +/− serologically active disease (Supplementary Figure S3) and correlated positively with disease activity measures including SLEDAI, erythrocyte sedimentation rate (ESR) and anti-double stranded DNA antibodies (dsDNA) and negatively with complement C3 (Figure 4B). Furthermore, atherogenic lipoproteins (ApoB, ApoB:A1 ratio, VLDL, IDL, and LDL) correlated positively, and atheroprotective lipoproteins (ApoA1 and HDL) correlated negatively, with lipid rafts in T-cells and B-cells (Figure 4C, Table S6), suggesting a role of lipoproteins in altering lymphocyte lipid metabolism and function. Interestingly, lipid raft expression in monocytes did not correlate with disease activity measures or lipoprotein profiles (Figure 4B–C).

To test whether serum lipid composition could alter immune cell lipid raft expression and potentially immune cell function, sera, and VLDL/LDL isolated from JSLE patients with clinically active and inactive disease and HCs were cultured with HC lymphocytes in vitro. Serum from JSLE patients with clinically active disease induced a significant increase in lipid rafts and activation markers in T-cells and B-cells compared to serum isolated from patients with low disease activity and HC’s (Supplementary Figure S4). This effect was recapitulated when isolated VLDL/LDL fractions from patients with clinically active and inactive disease were compared (Figure 4D).

**Figure 5:**
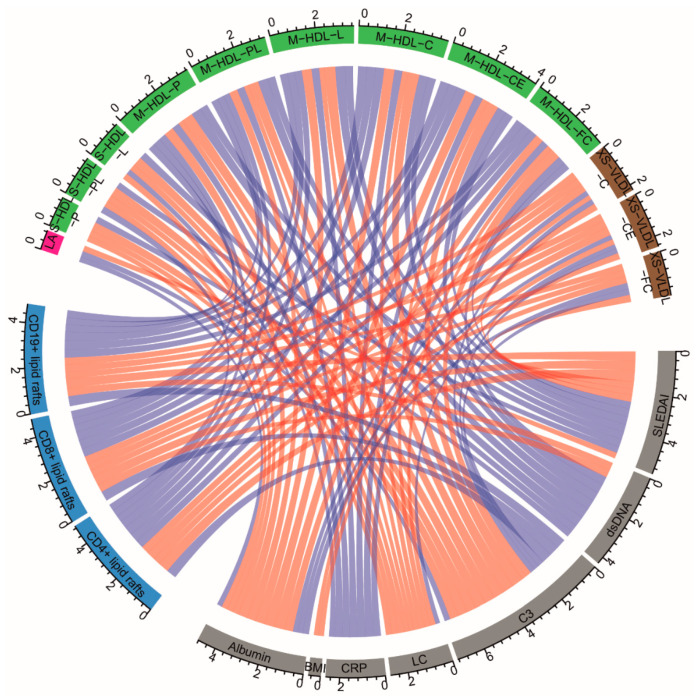
Association between selected serum lipid metabolites, clinical measure of disease activity and lipid rafts in JSLE. Correlations between selected lipid metabolites, clinical factors, and lipid raft. Pearson correlation coefficients are represented as edges connecting the lipid metabolites section (green/pink/brown), clinical factor (grey) section and lipid raft section (blue). The width of the edges represents the size of the correlation. Only statistically significant correlations following adjustment for multiple comparisons (10% FDR) are shown. Red lines represent positive correlation and blue lines represent negative correlation. Abbreviations: VLDL, very low-density lipoprotein; HDL, high density lipoprotein; XS, very small; S, small; M, medium; C, cholesterol; CE, cholesterol ester; FC, free cholesterol; L, total lipids; P, particles; PL, phospholipids; LA, linoleic acid; BMI, Body Mass Index; SLEDAI, Systemic Lupus Erythematosus Disease Activity Index; dsDNA, anti-double-stranded-DNA antibodies; C3, complement component 3; LC, lymphocyte count.

Thus atherogenic dyslipidaemia in JSLE patients is exacerbated in clinically active disease and may play a role in driving altered lipid raft expression and immune cell function. When JSLE is quiescent, forward lipid transport via VLDL/LDL is reduced, potentially lowering lipid accumulation in the periphery, and reducing both inflammation and cardiovascular risk through atherosclerosis. A correlative summary of the key lipids associated with JSLE and clinical measures of disease activity are summarised in Figure 5.

## 3. Discussion

This study provides an in-depth analysis of dyslipdaemia in a large cohort of adolescent patients with JSLE compared to age matched healthy donors. Three key points arise from this work; firstly, NMR metabolomics identified early dyslipidaemia in JSLE patients despite no significant difference observed in the lipid profile routinely used in clinical care, which was characterised by reduced small HDL subsets; secondly, increased clinical disease activity in JSLE exacerbated reductions in HDL subsets and was also associated with elevated atherogenic lipoprotein subsets compared to age-matched healthy donors; and finally, VLDL/LDL lipoprotein levels correlated positively with T-cell and B-cell plasma membrane lipid rafts and in vitro culture of VLDL/LDL isolated from patients with clinically active disease increased lymphocye lipid raft expression. These findings point to a relationship between systemic lipoprotein metabolism, immune cell plasma membrane lipids, and disease activity in patients with JSLE. This highlights a need for more targeted lipid metabolomic approaches beyond the standard clinical panel to detect dyslipidaemia in JSLE as well as investigation of more targeted strategies to address the dysregulated atherogenic lipid profile associated with JSLE in adolescence.

The majority of JSLE patients included in this study had standard clinical serum lipid measures within normal ranges for our routine clinical service. This is in contrast to multiple studies reporting HDL-cholesterol levels outside of the normal ranges for greater than 24% of the JSLE study patient cohort and/or significantly reduced levels in patients compared to HCs [11,29,30,31], and can most likely be explained by the almost universal use of hydroxychroloquine treatment in this JSLE cohort, which is known to reduce HDL-cholesterol levels [32], and patients having mostly mildly-moderate clinical activity. However, using an in-depth NMR metabolomic approach, this cohort of JSLE patients were characterised by globally lower small HDL subset concentrations (specifically small HDL particles, total lipid and phopholipid content) compared to age-matched HCs, regardless of disease activity status. This finding supports a fundamental change in lipid metabolism early in disease development that is resistant to current therapies. Changes in HDL composition have been described in SLE patients more generally [33], this includes increased triglycerides and serum amyloid A and reduced HDL-cholesterol:ApoA1 ratio and cholesterol esters and [33] paraoxinase-1 activity (an enzyme associated with HDL that reduces HDL and LDL oxidation) [34]. Paraoxinase activity can be blocked by anti-ApoA1 antibodies and contribute to the formation of proinflammatory HDL, a dysfunctional HDL modified by inflammation and characterised by reduced cholesterol exflux and reduced anti-oxidant capability [35,36]. In one study, 48.2% of women with SLE were found to have proinflammatory HDL [10], where the antioxidant capacity of atheroprotective HDL is lost, thus contributing to atherosclerosis. However, while this current study confirms a reduction in small-medium HDL concentrations, we did not see changes in cholesterol ester, triglyceride, or anti-ApoA1 antibody levels, most likely because most studies have been performed on adult SLE cohorts.

JSLE patients with clinically active disease had an exclusive significant increase in small and very small VLDL particles subsets, concentrations, and lipid composition as well as an exacerbated decrease in small HDL particles. VLDL particles contain more triglycerides compared to LDL and IDL [37], and previous reports show an association between increased triglycerides and disease activity in JSLE [11]. In contrast, a previous study in adult patients showed that there were no significant differences in traditional CVD-risk factors, such as the Framingham score incorporating cholesterol and LDL-C, between patients high and low disease activity [38]. Despite this, these patients with a high disease activity had early-stage, clinically silent diastolic dysfunction, supporting a role for echocardiography in CVD-risk assessments. Reduced HDL has also been reported in patients with adult SLE with high disease activity [11,29]. We also identified an increase in the ApoB:ApoA1 ratio exclusive to active patients; likely explained by increased ApoB-expressing small VLDL and decreased ApoA1-expressing small HDL in JSLE patients with more active disease. Importantly, this biomarker has been identified by previous studies to predict of CVD-risk in young people, including prospective studies showing that the expression of apolipoproteins in healthy children reflects subclinical atherosclerosis development [39] and cardiometabolic risk [40] in adulthood, and another study showing that the ApoB:ApoA1 ratio was associated with cardio-metabolic risk in a subset of JSLE patients [41]. Thus, a combination between early lipid biomarkers (such as those identified by our study), combined with traditional cardiovascular assessment tools (such as echocardiography), and measures of JSLE disease activity, could provide a more detailed analysis of CVD-risk in young patients for early clinical intervention. This could be incorporated into a clinical setting through regular and more detailed assessment of lipids in patients with persistently high disease activity, followed by cardiovascular scans for direct assessment of atherosclerosis. Early intervention would reduce long-term complications and mortality rates from CVD in patients with JSLE.

The influence of lipoproteins on T-cell and B-cell membrane lipids and immune cell function is not well understood but a growing body of evidence supports a role for lipid metabolism in immune cell function [12,13,36,42,43]. Here we found that T-cell and B-cell lipid rafts correlated positively with ApoB:A1, VLDL, LDL, and IDL but negatively with ApoA1 and HDL. Cholesterol efflux via ApoA1/HDL can alter immune cell function, including inhibiting macrophage foam cell formation associated with inflammation and atherogenesis [4], and inhibiting the ability of antigen presenting cells to stimulate T-cells [44]. In our study, HDL and ApoA1 correlated negatively with lipids associated with plasma membrane lipid rafts (cholesterol, PG, and PC [45]) in patients with more clinically active disease, suggesting that lipid efflux from immune cells could be defective in JSLE. A combination of increased lipid uptake via VLDL, IDL, and LDL and decreased lipid efflux to ApoA1/HDL may account for the increased membrane lipid raft expression in high disease activity patients. This hypothesis has also been suggested in metabolomic studies of multiple sclerosis [46]. In support, we showed that both serum and isolated VLDL and LDL from JSLE patients with active disease increased the level of lipid rafts in T-cells and B-cells associated with increased immune activation. Importantly, immune cell lipids are now recognised as targets for immunotherapies in cancer; inhibition of acetyl-CoA acetyltransferase 1 (ACAT1, cholesterol esterification enzyme that increases immune cell cholesterol levels) improves the efficacy of anti-PD1 therapy in melanoma and antiviral activity against hepatitis B due to a specific increase in CD8+ T-cell effector function against melanoma growth through lipid raft associated T-cell receptor (TCR) clustering and signalling [28,47]. Resistance to therapy in patients with ER^+^ breast cancer is also associated with upregulated cholesterol biosynthesis enzymes which is reversed experimentally by targeting lipid biosynthesis [48]. Similar mechanisms could therefore improve the efficacy of immune cell membrane receptor targeted biologics such as Rituximab (B-cells, anti-CD20) in SLE. Membrane lipid changes could also impact immune function via increased recruitment of soluble transport proteins, such as chloride intracellular channel (CLIC) proteins, from the aqueous phase into the immune cell membrane via lipid mediated processes [49]; these channels have been shown to increase proinflammatory cytokine expression [50].

Recognising and targeting dyslipdaemia at a younger age could be an opportunity to manage the accelerated atherosclerotic process and prevent future metabolic complications associated with JSLE, comorbidities or drug toxicity, future worsening of dyslipidaemia, flares, and atherosclerotic development in JSLE patients. Our previous work demonstrates that clinically approved drugs, statins (known to reduce circulating cholesterol) and inhibitors of glycolipid biosynthesis (miglustat) can reverse SLE-associated immune cell defects in vitro [16,17]. These effects were achieved selectively via reduction of membrane lipids, raising the possibility that targeting membrane lipids could control immune cell activation and may be a novel therapeutic target for autoimmunity. There is also some emerging evidence that targeting dyslipidaemia with statins in SLE is associated with decreased risk of flares [51]. Various studies have trialed the use of statins in SLE with mixed outcomes; some showing beneficial outcomes [52,53,54,55], and some not meeting their target such as the Lupus Atherosclerosis Prevention Study (LAPS) [56] in adults with SLE and the Atherosclerosis Prevention in Paediatric Lupus Erythematosus (APPLE) trial [57] in JSLE. The poor outcome of these trials was likely due to patient heterogeneity and unsuitable primary outcome measures; the future of these will depend on correct stratification of patients [58,59]. In support, we have previously reported that a lipoprotein stratification method could be key to the therapeutic management of dyslipidaemia in JSLE [60]. We identified the ApoB:ApoA1 ratio as a potential predictive biomarker of increased CVD risk in patients, who shared a serum metabolomic profile with adult SLE patients with sub-clinical atherosclerosis and a T-cell phenotype with T-cells isolated from human atherosclerotic plaque. These patients also had a more active disease trajectory over 5 years of follow up. Together with this work, these studies could improve clinical trial design and help identify patients that could benefit from more tailored disease or lipid-modifying therapy. Using these specific serum lipid biomarkers to stratify patients for lipid modifying therapeutic intervention, or retrospectively as a baseline from previously completed trials, could unmask the individual impact observed from the intervention in established outcome measures of cardiometabolic-risk. Future therapies based on replacing defective HDL are also showing promise in clinical trials [61].

In addition to current clinically prescribed therapies, increased dietary intake of omega-3 fatty acids has also shown therapeutic promise for reducing CVD risk in (J)SLE. A study in JSLE induced an increase in HDL-C [62] and another study in adult SLE showed decreased serum triglycerides and VLDL-cholesterol and increased HDL-cholesterol [63]. A diet intervention study in paediatric SLE patients with dyslipoproteinemia resulted in a significant decrease in serum triglyceride concentrations over 6 weeks which was reduced further by the addition of fish oils for a further 6 weeks [64]. Interestingly, our study showed that linoleic acid and total omega-6 fatty acids were exclusively decreased in JSLE patients with low disease activity compared to healthy controls, suggesting that the dietary balance between omega-3 and omega-6 fatty acids could be an important target in controlling JSLE CVD risk. Dietary lipids can also affect lipid raft composition and raft associated downstream signalling [65], whereby incorporation of the dietary PUFA, DHA, into T-cell membranes alters plasma membrane phospholipid expression and the localisation of immunogenic receptors, such as IL-2-receptor and Fc-receptors, into lipid raft microdomains. This modifies T-cell activation signals and may result in a more inflammatory and atherogenic local response.

This study had some limitations. The retrospective nature of this study also did not allow us to confidently clarify any cause–effect relationship, adding limitations to our findings. In addition, although we adjusted our analysis for race, more detailed racial descriptions, beyond the groups stated in the results, were not accessible for this study cohort. The vast majority of these patients had quiescent disease or only mild-moderate clinical activity, which is usually the case in the modern era, when patients have better access to treatments for JSLE. This meant that the threshold used for splitting the JSLE patients in two group was based on evidence of at least mild clinical activity (associated with serological activity, SLEDAI score of 4 or more), along with the young age of the patients, could explain the lower accuracy of the BRF model compared to that observed in adult patients with greater disease duration and treatment burden [66]. Despite this, we identified novel biomarkers of atherogenic dyslipidaemia in JSLE along with profound and highly significant alterations of lipoprotein metabolism associated with disease activity. With this respect, these patients were on various treatment regimens for their disease management. This, however, was adjusted for in a sub-analysis to confirm that the metabolic differences observed were not confounded by treatment. Another limitation of this study was the sex ratio difference between the HC and JSLE cohorts as JSLE is a disease with pronounced female bias. To account for this, we adjusted for demographic features in our BRF and logistic regression analysis. It was crucial that we performed this adjustment as we have shown previous sex differences in lipoprotein metabolism between men and women [67]. The use of these metabolites as biomarkers would need to be validated in separate cohorts and potentially in future clinical trials. As a preliminary estimation for this next research step, a post-hoc power calculation was performed on the levels of S-HDL-P (HC vs. JSLE) and XS-VLDL-FC (JSLE patients with high vs. low disease activity), providing an estimated validation sample size of 16 and 74 per group (*p* < 0.05, power = 80%) respectively to observe significant differences in expression. Finally, it was beyond the scope of this study to assess the functional and therapeutic implications of the lipoprotein induced lipid raft alterations and it will be important for future studies, including clinical trials and/or use of experimental models, to address this.

Together, we present here a case that lipid modification strategies could benefit JSLE patients to target both dyslipidaemia and inflammation, two major contributors to atherosclerosis and CVD. Reducing forward lipoprotientransport from the liver to the periphery (VLDL/IDL/LDL/ApoB) and increasing reverse(HDL/ApoA1) lipoprotein transport (from the periphery to the liver) could reduce the influence of dyslipidaemia on the membrane lipids and function of T-cells and B-cells and improve the efficacy of current disease specific therapeutics and of those currently in clinical trials. This work will hopefully lead to the development of better tailored guidelines and strategies to test and make use of the available lipid modifying therapies, as well as develop new ones for patients with JSLE to improve long-term disease outcomes and quality of life. There is, however, a need to test and validate these findings in a longitudinal clinical trial setting an in experimental models before influencing clinical guidelines.

## 4. Materials and Methods

### 4.1. Patients and Control Samples

This is a retrospective cohort study, where peripheral blood was collected from patients attending a young adult or adolescent rheumatology clinic at University College London Hospital (UCLH) or Great Ormond Street Hospital (GOSH) respectively. Teenage and young adult HC blood was collected at the Rayne building, UCL, from volunteers taking part in educational events such as young scientist days. Informed written consent was acquired from both patients and healthy controls under the ethical approval reference: REC11/LO/0330. All information was stored as anonymised data. Demographic information (including age, sex, and broad race categories), detailed clinical characteristics and disease features were recorded from patient files and questionnaires (Table 1). Disease activity was calculated using SLEDAI; due to the low average disease activity of this well clinically controlled cohort, a SLEDAI score of 4 or more was used to indicate clinically active disease [20]. Patients with SLEDAI < 4 were included in the clinically inactive group only if their disease was quiescent and the only elements of activity on the SLEDAI score were increased dsDNA antibodies +/− decreased complement C3 fraction which reflected only serological activity. Non-alcoholic fatty liver disease fibrosis scores were calculated using patient age, body mass index, platelet count, and measures of liver function [25]. Patients treated with rituximab and cyclophosphamide within the past year were excluded from the study due to previously reported substantial improvements in lipid measures following effective treatment [68].

### 4.2. Metabolomics

Serum from JSLE patients and HCs were analysied using the Nightingale nuclear magnetic resonance metabolomics platform (https://nightingalehealth.com/, Accessed on 18 February 2020) which includes over 130 blood lipid metabolic biomarkers (Supplementary Table S1). Included in the platform is the simultaneous measurement of routine lipid measures and in-depth lipoprotein measurements, such as particle size and content.

### 4.3. Metabolomics Data Analysis

The balanced random forest (BRF) approach was used with the randomForest package in R [69]. As the original sample set had an unbalanced HC:JSLE (32:65) ratio, the balanced method was applied in the bootstrap dataset construction, comparing equal numbers of cases and controls at each split. A parameter-tuning test was performed to maximise the model performance and 10,000 decision trees were used for model construction to ensure the reliable predictive performance of the model. Samples that were not included in the bootstrap dataset were termed the ‘out-of-bag’ (OOB) dataset and were used to validate the model performance. Demographic factors were included into the BRF model for adjustment purposes. For model performance evaluation, the receiver operator characteristic (ROC) plot and the area under the curve (AUC) of each model was computed with the pROC package in R [70]. Logistic regression was performed in RStudio [71] on each individual metabolomic biomarker adjusted for demographic (age, sex, race) and/or treatments (Table 1). Logistic regression analysis was visualised using the R package foresplotNMR [72]. 10-fold cross validation was applied to assess model overfitting with the R package caret [73].

### 4.4. Flow Cytometry

PBMCs (1 × 10^6^) were stained with fixable blue dead cell stain (ThermoFisher, Waltham, Massachusetts and USA) or Zombie NIR™ Fixable Viability Kit (Biolegend, London, UK). This was followed by washes and surface marker staining with antibodies CD4-BUV395, CD8-AF700, CD19-AF488, and CD14-BV711 (Biolegend, London, UK) followed by subsequent washes. Following surface staining, to determine the expression of membrane lipid rafts cells were subsequently stained with a surrogate lipid raft marker CTB^16^, conjugated to FITC (1:100 dilution in PBS) (Sigma, Dorset, UK) followed by subsequent washes and fixation in 2% PFA before running on the flow cytometer. Altneratively, cells were fixed filipin complex (Sigma) as described previously [74]. Stains and washes were carried out in cell staining buffer (Biolegend). Appropriate unstained controls were used for lipid stains.

Data was acquired using a LSRFORTESSA X-20 (BD, Wiltshire, UK) flow cytometer and analysis performed using FlowJo Single Cell Analysis Software (TreeStar). Application settings were created and Cytometer Setup and Tracking (CS&T) (BD) beads were run to assess cytometer performance for each experiment.

### 4.5. VLDL Isolation and Cell Culture

VLDL was isolated from pooled serum from patients and HCs using the using LDL/VLDL Purification Kit (Ultracentrifugation Free, CELL BIOLABS, San Diego, California, USA) according to manufacturer’s instructions. Purified LDL/VLDL was dialysed in PBS in Amicon Ultra centrifugal filter units (MILLIPORE, Hertfordshire, UK). A Pierce™ Bicinchoninic Acid assay (BCA) Protein Assay Kit (ThermoFisher) was used to quantify the isolated LDL/VLDL (Apolipoprotein B protein expression). Isolated lipoproteins were filtered through a 0.22-μm filter and stored at 4 °C for a maximum of 6 weeks. 1 × 10^6^ PBMCs were cultured with 10% isolated VLDL (concentration proportionate to physiological levels of the original pooled serum groups) in RPMI-1640 medium 48 h followed by lipid analysis by flow cytometry.

### 4.6. Statistical Analysis

Statistical analysis was performed using GraphPad Prism 7 software (https://www.graphpad.com/, Accessed on 9 November 2021). Data was tested for normal distribution using Kolmogorov–Smirnov test and parametric or non-parametric *t*-tests or correlations were used accordingly. Unpaired or paired *t*-tests (following tests for normal distribution) were used to compare ex-vivo patient/HC data or longitudinal matched sample/experimental data respectively. ROC curve analysis was performed using GraphPad Prism 7. Linear regression was performed with a 95% confidence interval used to calculate significance (Pearson’s correlation). MultiExperiment Viewer (MeV) was used to produce heat maps and perform hierarchical clustering on correlation data sets. Multiple testing was accounted for using false discovery rate correction (Benjamini, Krieger, and Yekutieli approach) of *p*-value thresholds on phenotyping and metabolomics data. Chord plots were produced using the ‘circlize’ package to summarise correlations (Pearson correlation coefficients, 10% FDR) between selected lipid metabolites, clinical factors and lipid rafts [75].

## Figures and Tables

**Table 1:** Demographic and clinical table of all patients and healthy donors.

	HC	JSLE	Clinically Inactive JSLE (+/− Serological Activity)	Clinically Active JSLE	*p*-Value
Total number	32	65	43	22	−
Female:Male	17:15	53:12	36:7	17:5	0.0086
Age, mean (range)	19 (16–25)	19.3 (13–25)	19.3 (13–24)	19.2 (14–25)	0.8436 *
BMI, median (IQR)	23.1 (20–24.5)	22.4 (20.3–26.7)	22.4 (20.3–26.6)	22.5 (20.4–27.1)	0.5416 *
Race, number (%)					
White	14 (44)	15 (23)	11 (26)	4 (18)	0.1141
Asian	10 (31)	27 (42)	18 (42)	9 (41)	0.7667
Black	1 (3)	17 (26)	11 (26)	6 (27)	0.0447
Other/unknown	5 (16)	6 (9)	4 (9)	2 (9)	0.7726
Disease characteristics, mean (range)					
Age of diagnosis	−	12.1 (0–18)	12.2 (2–17)	11.9 (0–18)	0.9676 *
Disease duration	−	7.0 (0–21)	6.8 (2–21)	7.3 (0–17)	0.9119 *
SLEDAI	−	2.5 (0–10)	0.8 (0–2)	5.8 (4–10)	<0.0001 *
Organ involvement (historical), n (%)					
Neurological	−	12 (18)	9 (21)	3 (14)	0.7732
Serositis	−	8 (12)	4 (9)	4 (18)	0.5877
Cutaneous	−	56 (86)	39 (91)	17 (77)	0.3331
Haematological	−	28 (43)	17 (40)	11 (50)	0.7225
Musculoskeletal	−	53 (82)	36 (84)	17 (77)	0.8179
Renal	−	20 (31)	10 (23)	10 (45)	0.1857
Serology, median (IQR)					
dsDNA (IU/mL) (NR = <50)	−	23 (3–141.5)	8 (2–40)	326 (88.5–587)	0.1116 *
Positive ENA (number, %)	−	40 (62)	26 (60)	14 (64)	0.9696 *
CRP (mg/L) (NR < 10)	−	1 (0.6–2.7)	1.1 (0.6–2.35)	0.7 (0.6–3.55)	0.7148 *
C3 (g/L) (NR = 0.9–1.8)	−	0.98 (0.74–1.18)	1.1 (0.92–1.2)	0.76 (0.58–0.97)	0.0009 *
LC (10^9^/L) (NR = 1.3–3.5)	−	1.24 (1.2–1.7)	1.37 (1.03–2.02)	1.19 (0.93–1.44)	0.0634 *
Albumin (g/L) (NR = 34–48)	−	42.3 (40.0–44.5)	42.7 (40.6–45.4)	42.2 (33.9–44.5)	0.0194
Clinical lipids, median (IQR)					
Cholesterol (NR < 5mmol/L)	−	4.0 (3.4–4.4)	3.9 (3.4–4.5)	4.0 (3.45–4.3)	0.6686 *
Cholesterol, number (% outside NR)	−	5.0 (8)	3 (7)	2 (9)	0.9552
Triglycerides (NR < 3mmol/L)	−	0.8 (0.53–1.15)	0.7 (0.5–1.0)	0.9 (0.65–1.7)	0.6034 *
Triglycerides, number (% outside NR)	−	4 (6)	2 (5)	2 (9)	0.7801
HDL-C (NR > 1mmol/L)	−	1.5 (1.2–1.7)	1.5 (1.2–1.7)	1.4 (1.15–1.6)	0.4333 *
HDL-C, number (% outside NR)	−	5 (8)	3 (7)	2 (9)	0.9552
LDL-C (NR < 3mmol/L)	−	2.1 (1.6–2.4)	2 (1.5–2.4)	2.2 (1.65–2.45)	0.5229 *
LDL-C, number (% outside NR)	−	6 (9)	4 (9)	2 (9)	0.9996
Current treatment, n (%)					
Hydroxychloroquine	−	60 (92)	39 (91)	21 (95)	0.7930
Mycophenolate mofetil	−	25 (38)	15 (35)	10 (45)	0.7092
Prednisolone	−	31 (48)	19 (44)	12 (55)	0.7312
Vitamin D	−	12 (18)	7 (16)	5 (23)	0.8179
Methotrexate	−	6 (9)	5 (12)	1 (5)	0.6468
Azathioprine	−	15 (23)	6 (14)	9 (41)	0.0509
Rituximab in the last year	−	0 (0)	0 (0)	0 (0)	−

## Data Availability

The data presented in this study are available in supplementary Tables S2–S4.

## Supplementary Materials

The following are available online at https://www.mdpi.com/article/10.3390/metabo12010003/s1, Table S1: List of Metabolic biomarkers. Table S2: Assocition between anti-ApoA1 antibodies and HDL metabolites in JSLE. Supplementary Table S3: Normalised logistic regression metabolomics data comparing patients with active and inactive JSLE to healthy controls. Supplementary Table S4: List of metabolites that significantly correlate with disease activity (SLEDAI) scores in JSLE. Supplementary Table S5: Metabolites significantly associated with a disease flare in JSLE. Supplementary Table S6: Pearson’s correlation coefficients and *p* values from the correlation between JSLE patient lipid raft expression on immune cell subsets and serum lipids (metabolomics). Figure S1: Additional top features driving JSLE from HCs the balanced random forest model. Figure S2: Atherogenic lipoproteins are associated with increased disease activity in JSLE. Supplementary Figure S3: Immune cell lipid raft expression in JSLE patients. Supplementary Figure S4: Immune cell lipid metabolism is stimulated by in vitro culture with serum from JSLE patients with active disease.
