# Supplementary material for: Metabolomics Defines Complex Patterns of Dyslipidaemia in Juvenile-SLE Patients Associated with Inflammation and Potential Cardiovascular Disease Risk
Source: Metabolites. 2021 Dec 21;12(1):3. doi: 10.3390/metabo12010003 (PMC8779263; doi:10.3390/metabo12010003)
Supplement: Supplementary file 1 [file metabolites-12-00003-s001.zip › metabolites-1524139-suppl conversion.pdf]

Metabolomics defines complex patterns of dyslipidaemia in juvenile-SLE patients associated with inflammation and potential cardiovascular disease risk

Supplemental Information

Fatty acids:	L-VLDL-TG	LDL:	HDL:	Lipoprotein size:
TotFA	M-VLDL-P	L-LDL-P	XL-HDL-P	VLDL-D
UnSat	M-VLDL-L	L-LDL-L	XL-HDL-L	LDL-D
DHA	M-VLDL-PL	L-LDL-PL	XL-HDL-PL	HDL-D
LA	M-VLDL-C	L-LDL-C	XL-HDL-C	Total cholesterol:
FAw3	M-VLDL-CE	L-LDL-CE	XL-HDL-CE	Serum-C
FAw6	M-VLDL-FC	L-LDL-FC	XL-HDL-FC	VLDL-C
PUFA	M-VLDL-TG	L-LDL-TG	XL-HDL-TG	LDL-C
MUFA	S-VLDL-P	M-LDL-P	L-HDL-P	HDL-C
SFA	S-VLDL-L	M-LDL-L	L-HDL-L	HDL2-C
VLDL:	S-VLDL-PL	M-LDL-PL	L-HDL-PL	HDL3-C
XXL-VLDL-P	S-VLDL-C	M-LDL-C	L-HDL-C	EstC
XXL-VLDL-L	S-VLDL-CE	M-LDL-CE	L-HDL-CE	FreeC
XXL-VLDL-PL	S-VLDL-FC	M-LDL-FC	L-HDL-FC	Total triglycerides:
XXL-VLDL-C	S-VLDL-TG	M-LDL-TG	L-HDL-TG	Serum-TG
XXL-VLDL-CE	XS-VLDL-P	S-LDL-P	M-HDL-P	VLDL-TG
XXL-VLDL-FC	XS-VLDL-L	S-LDL-L	M-HDL-L	LDL-TG
XXL-VLDL-TG	XS-VLDL-PL	S-LDL-PL	M-HDL-PL	HDL-TG
XL-VLDL-P	XS-VLDL-C	S-LDL-C	M-HDL-C	Phospholipids:
XL-VLDL-L	XS-VLDL-CE	S-LDL-CE	M-HDL-CE	TotPG
XL-VLDL-PL	XS-VLDL-FC	S-LDL-FC	M-HDL-FC	TG/PG
XL-VLDL-C	XS-VLDL-TG	S-LDL-TG	M-HDL-TG	PC
XL-VLDL-CE	IDL:		S-HDL-P	SM
XL-VLDL-FC	IDL-P		S-HDL-L	TotCho
XL-VLDL-TG	IDL-L		S-HDL-PL	Apolipoproteins:
L-VLDL-P	IDL-PL		S-HDL-C	ApoA1
L-VLDL-L	IDL-C		S-HDL-CE	ApoB
L-VLDL-PL	IDL-CE		S-HDL-FC	ApoB/ApoA1
L-VLDL-C	IDL-FC		S-HDL-TG	
L-VLDL-CE	IDL-TG			
L-VLDL-FC				

Table S1: List of Metabolic biomarkers: Nightingale Health metabolomics platform (https://nightingalehealth.com/) was used to measure biomarkers in JSLE patient serum. This service measures blood metabolic biomarkers using nuclear magnetic resonance (NMR) spectroscopy. The platform can simultaneously measure amino acids, fatty acids, glycolysis metabolites, routine lipid measures (mmol/l), apolipoproteins (g/l) and in depth lipoprotein measurements such as particle size (nm), concentration and lipid content (mmol/l). The platform provides repeatable measurements with no batch effects. The Nightingale Health service has been thoroughly validated and has been used to measure over 500,000 samples from both research and clinical trials.
Abbreviations: Apo, apolipoprotein; VLDL, very low density lipoprotein; IDL, intermediate density lipoprotein; LDL, low density lipoprotein; HDL, high density lipoprotein; XXL-VLDL, chylomicrons and extremely large VLDL; X-Large, very large; X-small, very small; Est (esterified), PG (Phosphoglyceride), PC, Phosphatidylcholine; SM, Sphingomyelins; Unsat, Unsaturated; DHA, Docosahexaenoic acid; LA, Linoleic acid; FAw3, Omega-3 fatty acids; FAw6, Omega-6 fatty acids; PUFA, Polyunsaturated fatty acids; MUFA, Monounsaturated fatty acids; SFA, Saturated fatty acids; TG, triglycerides; PL, phospohlipids, FC, free cholesterol, C, cholesterol, CE, cholesterol esters; P, particle; L, lipid.

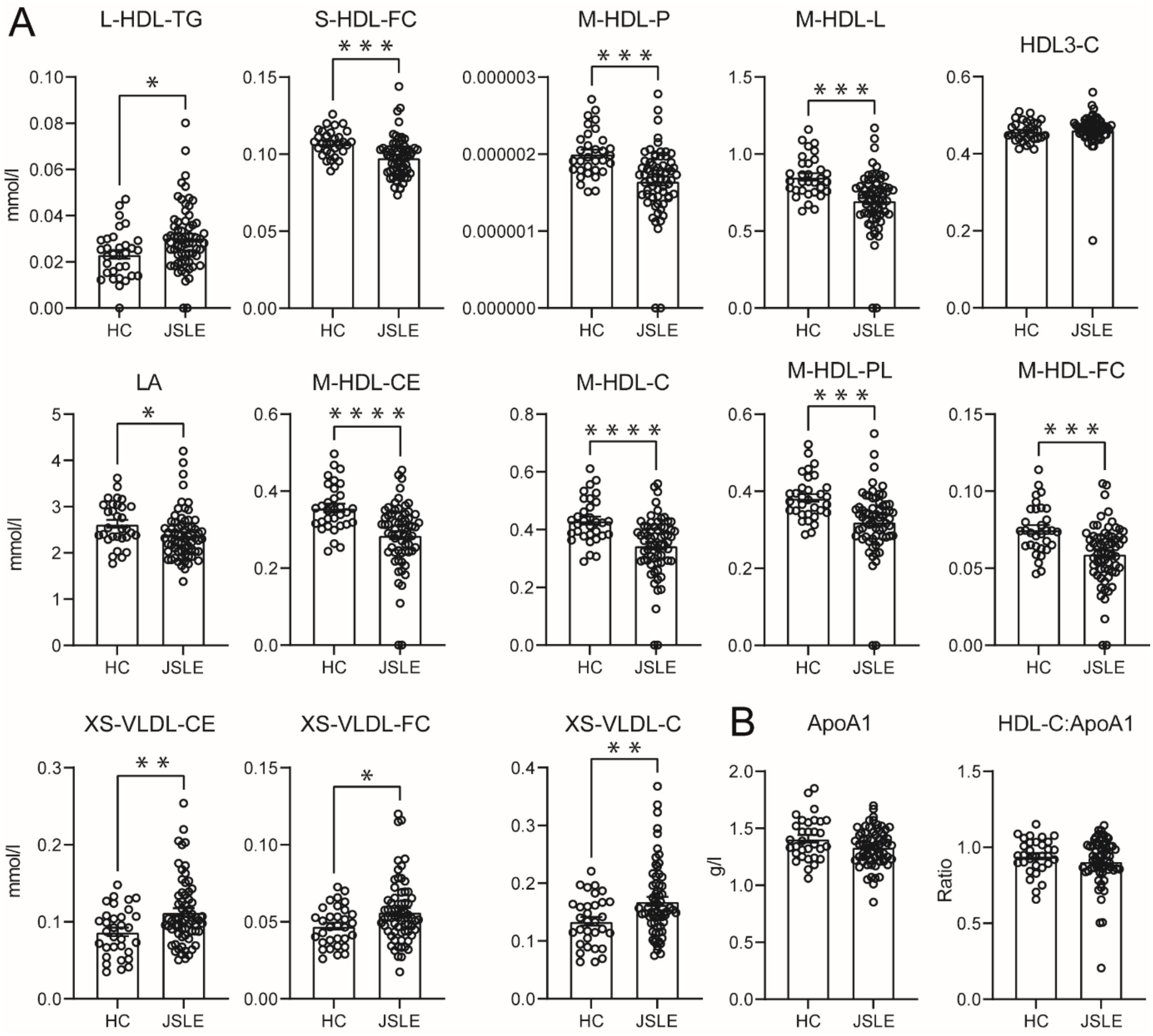

Figure S1: Additional top features driving JSLE from HCs the balanced random forest model. Individual scatter plots of **(A)** the top metabolites from the BRF predictive model and **(B)** measures of ApoA1 comparing HCs (n=32) to JSLE patients (n=65). Unpaired t test. *=P<0.05, **=P<0.01, ***=P<0.001, ****=P<0.0001. ApoA1-IgG was detected as previously described [1].
Abbreviations: VLDL, very low density lipoprotein; HDL, high density lipoprotein; XS, very small; S, small; M, medium; L, large; C, cholesterol; CE, cholesterol ester; FA, fatty acid; FC, free cholesterol; L, total lipids; P, particles; PL, phospholipids; TG, triglycerides; LA, linoleic acid.

**Anti-ApoA1 vs.**	**S-HDL-P**	**S-HDL-L**	**S-HDL-PL**
Spearman (r value)	-0.1727	-0.1585	-0.1104
P value	0.3989	0.4394	0.5912

Table S2: Assocition between anti-ApoA1 antibodies and HDL metabolites in JSLE: Table displaying Spearman correlation coeficients (r values) and p values for the correlation between the top HDL metabolites from the BRF model and the levels of circulating anti-ApoA1 antibodies in isolated serum from matched patients with JSLE (n=26).

	HC vs SLE					HC vs Inactive JSLE					HC vs JSLE				
	Beta	Beta SE	Odds Ratio	Odds Ratio SE	p-value	Beta	Beta SE	Odds Ratio	Odds Ratio SE	p-value	Beta	Beta SE	Odds Ratio	Odds Ratio SE	p-value
ApoA1	-0.85	0.47	0.43	1.60	0.07	-0.42	0.31	0.43	1.60	0.18	-0.50	0.30	0.43	1.60	0.09
ApoB	0.68	0.37	1.96	1.45	0.07	-0.21	0.29	1.96	1.45	0.46	0.13	0.25	1.96	1.45	0.59
ApoB/ApoA1	1.02	0.49	2.77	1.63	0.04	-0.02	0.31	2.77	1.63	0.95	0.32	0.30	2.77	1.63	0.30
DHA	-0.21	0.36	0.81	1.44	0.57	-0.03	0.29	0.81	1.44	0.91	-0.05	0.27	0.81	1.44	0.86
EstC	0.46	0.37	1.59	1.44	0.21	-0.30	0.29	1.59	1.44	0.31	0.01	0.25	1.59	1.44	0.97
FAw3	0.14	0.33	1.15	1.39	0.67	0.14	0.31	1.15	1.39	0.66	0.15	0.28	1.15	1.39	0.59
FAw6	0.05	0.33	1.05	1.40	0.88	-0.66	0.31	1.05	1.40	0.03	-0.32	0.25	1.05	1.40	0.21
FreeC	0.66	0.39	1.94	1.48	0.09	-0.17	0.29	1.94	1.48	0.56	0.13	0.26	1.94	1.48	0.61
HDL-C	-0.95	0.47	0.39	1.61	0.05	-0.31	0.31	0.39	1.61	0.33	-0.46	0.30	0.39	1.61	0.13
HDL-D	-0.32	0.40	0.73	1.50	0.43	0.22	0.31	0.73	1.50	0.47	0.05	0.29	0.73	1.50	0.86
HDL-TG	0.53	0.40	1.69	1.49	0.18	0.15	0.31	1.69	1.49	0.64	0.26	0.31	1.69	1.49	0.40
HDL2-C	-1.02	0.47	0.36	1.60	0.03	-0.34	0.32	0.36	1.60	0.28	-0.51	0.30	0.36	1.60	0.10
HDL3-C	0.39	0.36	1.48	1.43	0.28	0.14	0.28	1.48	1.43	0.62	0.28	0.28	1.48	1.43	0.32
IDL-C	0.63	0.37	1.88	1.45	0.09	-0.13	0.27	1.88	1.45	0.62	0.14	0.24	1.88	1.45	0.57
IDL-CE	0.61	0.36	1.85	1.44	0.09	-0.15	0.27	1.85	1.44	0.58	0.12	0.24	1.85	1.44	0.61
IDL-FC	0.67	0.38	1.95	1.46	0.08	-0.08	0.27	1.95	1.46	0.77	0.18	0.25	1.95	1.46	0.48
IDL-L	0.69	0.38	2.00	1.46	0.07	-0.11	0.28	2.00	1.46	0.69	0.17	0.24	2.00	1.46	0.49
IDL-P	0.70	0.38	2.02	1.46	0.06	-0.11	0.28	2.02	1.46	0.69	0.17	0.25	2.02	1.46	0.48
IDL-PL	0.67	0.38	1.96	1.46	0.07	-0.13	0.27	1.96	1.46	0.65	0.16	0.24	1.96	1.46	0.52
IDL-TG	0.98	0.52	2.66	1.69	0.06	0.12	0.37	2.66	1.69	0.75	0.42	0.36	2.66	1.69	0.25
L-HDL-C	-0.67	0.42	0.51	1.52	0.11	0.03	0.30	0.51	1.52	0.92	-0.17	0.27	0.51	1.52	0.54
L-HDL-CE	-0.69	0.42	0.50	1.52	0.10	0.03	0.30	0.50	1.52	0.93	-0.17	0.27	0.50	1.52	0.52
L-HDL-FC	-0.61	0.42	0.54	1.52	0.14	0.04	0.30	0.54	1.52	0.88	-0.14	0.28	0.54	1.52	0.61
L-HDL-L	-0.73	0.43	0.48	1.53	0.09	0.01	0.30	0.48	1.53	0.97	-0.19	0.27	0.48	1.53	0.49
L-HDL-P	-0.73	0.43	0.48	1.53	0.09	0.01	0.30	0.48	1.53	0.96	-0.19	0.27	0.48	1.53	0.50
L-HDL-PL	-0.84	0.44	0.43	1.55	0.06	-0.08	0.30	0.43	1.55	0.80	-0.27	0.28	0.43	1.55	0.33
L-HDL-TG	0.23	0.40	1.26	1.49	0.57	0.64	0.35	1.26	1.49	0.07	0.51	0.32	1.26	1.49	0.11
L-LDL-C	0.66	0.37	1.94	1.45	0.07	-0.21	0.27	1.94	1.45	0.45	0.11	0.24	1.94	1.45	0.64
L-LDL-CE	0.66	0.37	1.94	1.45	0.07	-0.22	0.27	1.94	1.45	0.42	0.10	0.24	1.94	1.45	0.66
L-LDL-FC	0.66	0.37	1.93	1.45	0.08	-0.17	0.27	1.93	1.45	0.53	0.13	0.24	1.93	1.45	0.59
L-LDL-L	0.68	0.37	1.97	1.45	0.07	-0.20	0.27	1.97	1.45	0.46	0.12	0.24	1.97	1.45	0.62
L-LDL-P	0.69	0.38	1.98	1.46	0.07	-0.20	0.28	1.98	1.46	0.47	0.12	0.24	1.98	1.46	0.60
L-LDL-PL	0.60	0.36	1.82	1.44	0.10	-0.27	0.28	1.82	1.44	0.32	0.07	0.24	1.82	1.44	0.78
L-LDL-TG	0.93	0.48	2.53	1.61	0.05	0.14	0.36	2.53	1.61	0.70	0.42	0.34	2.53	1.61	0.22
L-VLDL-C	0.51	0.43	1.66	1.54	0.24	-0.27	0.30	1.66	1.54	0.37	-0.01	0.29	1.66	1.54	0.97
L-VLDL-CE	0.58	0.44	1.79	1.55	0.18	-0.26	0.30	1.79	1.55	0.40	0.02	0.29	1.79	1.55	0.94
L-VLDL-FC	0.42	0.42	1.52	1.52	0.32	-0.28	0.30	1.52	1.52	0.36	-0.05	0.29	1.52	1.52	0.88
L-VLDL-L	0.40	0.42	1.49	1.52	0.34	-0.34	0.30	1.49	1.52	0.26	-0.09	0.28	1.49	1.52	0.75
L-VLDL-P	0.39	0.42	1.48	1.52	0.35	-0.35	0.30	1.48	1.52	0.25	-0.10	0.28	1.48	1.52	0.73
L-VLDL-PL	0.42	0.42	1.53	1.52	0.31	-0.33	0.30	1.53	1.52	0.27	-0.08	0.28	1.53	1.52	0.78
L-VLDL-TG	0.35	0.42	1.42	1.52	0.40	-0.37	0.30	1.42	1.52	0.23	-0.12	0.28	1.42	1.52	0.66
LA	-0.14	0.35	0.87	1.41	0.68	-0.89	0.33	0.87	1.41	0.01	-0.50	0.26	0.87	1.41	0.06
LDL-C	0.69	0.37	2.00	1.45	0.07	-0.22	0.27	2.00	1.45	0.42	0.12	0.24	2.00	1.45	0.63
LDL-D	-0.10	0.42	0.90	1.51	0.80	0.84	0.40	0.90	1.51	0.04	0.48	0.33	0.90	1.51	0.15
LDL-TG	0.83	0.45	2.30	1.57	0.06	0.00	0.34	2.30	1.57	0.99	0.32	0.32	2.30	1.57	0.32
M-HDL-C	-3.06	1.03	0.05	2.80	0.003	-1.00	0.41	0.05	2.80	0.01	-1.35	0.43	0.05	2.80	0.002
M-HDL-CE	-3.16	1.06	0.04	2.90	0.003	-1.02	0.41	0.04	2.90	0.01	-1.39	0.45	0.04	2.90	0.002
M-HDL-FC	-2.71	0.91	0.07	2.50	0.003	-0.91	0.39	0.07	2.50	0.02	-1.18	0.40	0.07	2.50	0.003
M-HDL-L	-3.49	1.15	0.03	3.16	0.002	-0.94	0.39	0.03	3.16	0.01	-1.35	0.44	0.03	3.16	0.002
M-HDL-P	-3.60	1.19	0.03	3.27	0.002	-0.93	0.38	0.03	3.27	0.01	-1.35	0.44	0.03	3.27	0.002
M-HDL-PL	-3.25	1.10	0.04	2.99	0.003	-0.84	0.37	0.04	2.99	0.02	-1.23	0.42	0.04	2.99	0.004
M-HDL-TG	-0.64	0.41	0.53	1.51	0.12	-0.31	0.29	0.53	1.51	0.29	-0.35	0.27	0.53	1.51	0.19
M-LDL-C	0.72	0.38	2.05	1.46	0.06	-0.23	0.27	2.05	1.46	0.41	0.12	0.24	2.05	1.46	0.62
M-LDL-CE	0.71	0.38	2.04	1.46	0.06	-0.22	0.27	2.04	1.46	0.42	0.12	0.24	2.04	1.46	0.62
M-LDL-FC	0.73	0.38	2.08	1.46	0.06	-0.24	0.28	2.08	1.46	0.38	0.12	0.24	2.08	1.46	0.62
M-LDL-L	0.72	0.38	2.05	1.46	0.06	-0.25	0.28	2.05	1.46	0.38	0.12	0.24	2.05	1.46	0.63
M-LDL-P	0.72	0.38	2.05	1.46	0.06	-0.25	0.28	2.05	1.46	0.37	0.12	0.24	2.05	1.46	0.63
M-LDL-PL	0.63	0.36	1.87	1.44	0.08	-0.35	0.29	1.87	1.44	0.23	0.06	0.24	1.87	1.44	0.81
M-LDL-TG	0.79	0.43	2.21	1.54	0.07	-0.07	0.33	2.21	1.54	0.84	0.27	0.31	2.21	1.54	0.38
M-VLDL-C	0.66	0.44	1.93	1.55	0.13	-0.13	0.31	1.93	1.55	0.68	0.15	0.31	1.93	1.55	0.62
M-VLDL-CE	0.77	0.43	2.15	1.54	0.08	-0.01	0.32	2.15	1.54	0.98	0.27	0.31	2.15	1.54	0.38
M-VLDL-FC	0.50	0.43	1.66	1.53	0.24	-0.25	0.30	1.66	1.53	0.41	0.02	0.30	1.66	1.53	0.96
M-VLDL-L	0.52	0.43	1.67	1.54	0.23	-0.25	0.31	1.67	1.54	0.41	0.02	0.30	1.67	1.54	0.93
M-VLDL-P	0.50	0.43	1.65	1.54	0.24	-0.26	0.31	1.65	1.54	0.39	0.01	0.30	1.65	1.54	0.96
M-VLDL-PL	0.54	0.43	1.72	1.54	0.21	-0.24	0.31	1.72	1.54	0.43	0.04	0.30	1.72	1.54	0.88
M-VLDL-TG	0.43	0.42	1.54	1.53	0.31	-0.32	0.31	1.54	1.53	0.30	-0.04	0.29	1.54	1.53	0.88
MUFA	0.46	0.38	1.58	1.47	0.23	-0.34	0.29	1.58	1.47	0.25	-0.03	0.27	1.58	1.47	0.91
PC	0.28	0.36	1.32	1.44	0.44	-0.33	0.29	1.32	1.44	0.27	-0.10	0.26	1.32	1.44	0.70
PUFA	0.06	0.33	1.07	1.39	0.85	-0.53	0.29	1.07	1.39	0.07	-0.26	0.25	1.07	1.39	0.30
S-HDL-C	-0.57	0.48	0.56	1.62	0.24	-0.88	0.35	0.56	1.62	0.01	-0.68	0.33	0.56	1.62	0.04
S-HDL-CE	-0.16	0.43	0.85	1.54	0.71	-0.71	0.33	0.85	1.54	0.03	-0.44	0.30	0.85	1.54	0.15
S-HDL-FC	-1.62	0.55	0.20	1.74	0.003	-0.65	0.33	0.20	1.74	0.05	-0.81	0.32	0.20	1.74	0.01
S-HDL-L	-2.15	0.72	0.12	2.06	0.003	-1.11	0.39	0.12	2.06	0.00	-1.18	0.36	0.12	2.06	0.001
S-HDL-P	-2.11	0.71	0.12	2.02	0.003	-1.08	0.39	0.12	2.02	0.01	-1.14	0.36	0.12	2.02	0.002
S-HDL-PL	-2.59	0.84	0.07	2.31	0.002	-0.84	0.37	0.07	2.31	0.02	-1.07	0.36	0.07	2.31	0.003
S-HDL-TG	0.77	0.48	2.17	1.61	0.11	0.05	0.33	2.17	1.61	0.89	0.29	0.33	2.17	1.61	0.39
S-LDL-C	0.71	0.38	2.04	1.46	0.06	-0.25	0.28	2.04	1.46	0.37	0.11	0.24	2.04	1.46	0.65
S-LDL-CE	0.71	0.38	2.04	1.46	0.06	-0.24	0.27	2.04	1.46	0.39	0.12	0.24	2.04	1.46	0.63
S-LDL-FC	0.71	0.37	2.04	1.45	0.05	-0.29	0.29	2.04	1.45	0.33	0.09	0.25	2.04	1.45	0.73
S-LDL-L	0.67	0.37	1.95	1.44	0.07	-0.33	0.29	1.95	1.44	0.26	0.06	0.24	1.95	1.44	0.79
S-LDL-P	0.66	0.37	1.93	1.44	0.07	-0.34	0.29	1.93	1.44	0.24	0.06	0.24	1.93	1.44	0.81
S-LDL-PL	0.53	0.35	1.69	1.42	0.13	-0.53	0.33	1.69	1.42	0.11	-0.07	0.25	1.69	1.42	0.78
S-LDL-TG	0.56	0.37	1.75	1.45	0.13	-0.35	0.29	1.75	1.45	0.23	0.03	0.26	1.75	1.45	0.91
S-VLDL-C	0.87	0.41	2.38	1.51	0.04	0.06	0.31	2.38	1.51	0.84	0.36	0.30	2.38	1.51	0.22
S-VLDL-CE	0.92	0.40	2.52	1.50	0.02	0.18	0.31	2.52	1.50	0.56	0.43	0.29	2.52	1.50	0.13
S-VLDL-FC	0.75	0.44	2.12	1.56	0.09	-0.14	0.31	2.12	1.56	0.64	0.19	0.30	2.12	1.56	0.53
S-VLDL-L	0.75	0.45	2.12	1.57	0.09	-0.15	0.31	2.12	1.57	0.63	0.18	0.30	2.12	1.57	0.55
S-VLDL-P	0.73	0.45	2.08	1.57	0.11	-0.17	0.31	2.08	1.57	0.58	0.16	0.30	2.08	1.57	0.60
S-VLDL-PL	0.71	0.44	2.02	1.55	0.11	-0.20	0.31	2.02	1.55	0.51	0.14	0.29	2.02	1.55	0.63
S-VLDL-TG	0.61	0.46	1.84	1.58	0.18	-0.27	0.31	1.84	1.58	0.37	0.05	0.30	1.84	1.58	0.87

Serum-C	0.53	0.37	1.69	1.44	0.15	-0.28	0.29	1.69	1.44	0.33	0.04	0.25	1.69	1.44	0.86
Serum-TG	0.54	0.43	1.71	1.54	0.21	-0.23	0.30	1.71	1.54	0.44	0.04	0.30	1.71	1.54	0.89
SFA	0.44	0.35	1.55	1.42	0.22	-0.24	0.29	1.55	1.42	0.41	0.01	0.27	1.55	1.42	0.97
SM	0.64	0.40	1.89	1.49	0.11	-0.09	0.30	1.89	1.49	0.77	0.17	0.27	1.89	1.49	0.54
TG/PG	0.36	0.44	1.44	1.56	0.41	-0.21	0.32	1.44	1.56	0.50	-0.05	0.31	1.44	1.56	0.88
TotCho	0.48	0.38	1.61	1.46	0.21	-0.21	0.30	1.61	1.46	0.48	0.03	0.27	1.61	1.46	0.91
TotFA	0.34	0.34	1.40	1.40	0.32	-0.38	0.29	1.40	1.40	0.19	-0.09	0.26	1.40	1.40	0.74
TotPG	0.28	0.35	1.32	1.43	0.43	-0.25	0.29	1.32	1.43	0.39	-0.05	0.26	1.32	1.43	0.83
UnSat	-0.17	0.38	0.84	1.46	0.65	0.37	0.31	0.84	1.46	0.24	0.20	0.28	0.84	1.46	0.48
VLDL-C	0.82	0.43	2.27	1.53	0.06	0.04	0.32	2.27	1.53	0.89	0.33	0.31	2.27	1.53	0.29
VLDL-D	-0.08	0.41	0.92	1.50	0.84	-0.39	0.32	0.92	1.50	0.23	-0.28	0.28	0.92	1.50	0.32
VLDL-TG	0.46	0.43	1.58	1.53	0.28	-0.31	0.31	1.58	1.53	0.32	-0.03	0.29	1.58	1.53	0.93
XL-HDL-C	0.55	0.41	1.74	1.50	0.18	0.20	0.29	1.74	1.50	0.49	0.28	0.28	1.74	1.50	0.31
XL-HDL-CE	0.64	0.42	1.90	1.51	0.12	0.24	0.29	1.90	1.51	0.42	0.33	0.28	1.90	1.51	0.23
XL-HDL-FC	0.30	0.39	1.35	1.48	0.44	0.10	0.29	1.35	1.48	0.74	0.14	0.27	1.35	1.48	0.61
XL-HDL-L	0.27	0.40	1.31	1.50	0.50	0.20	0.30	1.31	1.50	0.50	0.22	0.29	1.31	1.50	0.44
XL-HDL-P	0.26	0.40	1.29	1.50	0.52	0.21	0.30	1.29	1.50	0.49	0.22	0.29	1.29	1.50	0.44
XL-HDL-PL	-0.04	0.40	0.96	1.49	0.92	0.18	0.31	0.96	1.49	0.56	0.12	0.29	0.96	1.49	0.68
XL-HDL-TG	0.64	0.39	1.89	1.48	0.11	0.34	0.32	1.89	1.48	0.30	0.40	0.30	1.89	1.48	0.19
XL-VLDL-C	0.54	0.44	1.71	1.55	0.22	-0.19	0.30	1.71	1.55	0.53	0.04	0.30	1.71	1.55	0.90
XL-VLDL-CE	0.61	0.46	1.85	1.58	0.18	-0.19	0.31	1.85	1.58	0.54	0.06	0.31	1.85	1.58	0.84
XL-VLDL-FC	0.44	0.41	1.55	1.51	0.29	-0.19	0.30	1.55	1.51	0.52	0.01	0.29	1.55	1.51	0.97
XL-VLDL-L	0.38	0.41	1.47	1.51	0.36	-0.28	0.30	1.47	1.51	0.35	-0.07	0.28	1.47	1.51	0.82
XL-VLDL-P	0.37	0.41	1.45	1.51	0.37	-0.29	0.30	1.45	1.51	0.33	-0.07	0.28	1.45	1.51	0.79
XL-VLDL-PL	0.39	0.41	1.48	1.50	0.33	-0.26	0.30	1.48	1.50	0.38	-0.04	0.28	1.48	1.50	0.88
XL-VLDL-TG	0.33	0.41	1.40	1.51	0.42	-0.32	0.30	1.40	1.51	0.29	-0.10	0.28	1.40	1.51	0.72
XS-VLDL-C	1.07	0.45	2.91	1.56	0.02	0.42	0.32	2.91	1.56	0.18	0.59	0.30	2.91	1.56	0.05
XS-VLDL-CE	1.03	0.44	2.80	1.55	0.02	0.46	0.32	2.80	1.55	0.15	0.60	0.30	2.80	1.55	0.04
XS-VLDL-FC	1.11	0.46	3.03	1.58	0.02	0.31	0.32	3.03	1.58	0.34	0.53	0.30	3.03	1.58	0.08
XS-VLDL-L	0.95	0.44	2.59	1.54	0.03	0.20	0.32	2.59	1.54	0.54	0.43	0.30	2.59	1.54	0.14
XS-VLDL-P	0.93	0.43	2.52	1.54	0.03	0.16	0.32	2.52	1.54	0.61	0.41	0.30	2.52	1.54	0.17
XS-VLDL-PL	0.82	0.40	2.27	1.50	0.04	-0.02	0.29	2.27	1.50	0.95	0.25	0.26	2.27	1.50	0.33
XS-VLDL-TG	0.81	0.50	2.25	1.66	0.11	-0.07	0.34	2.25	1.66	0.83	0.25	0.34	2.25	1.66	0.46
XXL-VLDL-C	0.60	0.44	1.82	1.55	0.17	-0.15	0.31	1.82	1.55	0.62	0.09	0.31	1.82	1.55	0.78
XXL-VLDL-CE	0.80	0.47	2.21	1.60	0.09	-0.11	0.32	2.21	1.60	0.73	0.19	0.32	2.21	1.60	0.56
XXL-VLDL-FC	0.34	0.40	1.41	1.49	0.39	-0.20	0.30	1.41	1.49	0.51	-0.03	0.29	1.41	1.49	0.91
XXL-VLDL-L	0.40	0.39	1.49	1.48	0.31	-0.28	0.30	1.49	1.48	0.36	-0.05	0.27	1.49	1.48	0.86
XXL-VLDL-P	0.39	0.39	1.48	1.47	0.31	-0.29	0.30	1.48	1.47	0.34	-0.06	0.27	1.48	1.47	0.84
XXL-VLDL-PL	0.35	0.38	1.42	1.46	0.36	-0.24	0.30	1.42	1.46	0.42	-0.04	0.27	1.42	1.46	0.88
XXL-VLDL-TG	0.36	0.38	1.43	1.46	0.35	-0.31	0.30	1.43	1.46	0.30	-0.08	0.27	1.43	1.46	0.77

Table S3: Normalised logistic regression metabolomics data comparing patients with active and inactive JSLE to healthy controls: Table displaying the beta and odds ratio values with SE and p values from the comparison of metabolite concentrations of total (n=65), active (n=22) and inactive (n=43) JSLE patients to healthy controls (HCs, n=32) by logistic regression analysis adjusted for age, sex and race. Significant values between groups are shown in red.

Metabolite	R (pearson)	P
XXL-VLDL-P	0.3008	0.0114
XXL-VLDL-L	0.2913	0.0144
XXL-VLDL-PL	0.272	0.0228
XXL-VLDL-C	0.3114	0.0087
XXL-VLDL-CE	0.3287	0.0055
XXL-VLDL-FC	0.2846	0.017
XXL-VLDL-TG	0.285	0.0168
XL-VLDL-P	0.3702	0.0016
XL-VLDL-L	0.3724	0.0015
XL-VLDL-PL	0.3769	0.0013
XL-VLDL-C	0.4078	0.0005
XL-VLDL-CE	0.4213	0.0003
XL-VLDL-FC	0.3854	0.001
XL-VLDL-TG	0.3532	0.0027
L-VLDL-P	0.3876	0.0009
L-VLDL-L	0.3895	0.0009
L-VLDL-PL	0.384	0.001
L-VLDL-C	0.4011	0.0006
L-VLDL-CE	0.4098	0.0004
L-VLDL-FC	0.3893	0.0009
L-VLDL-TG	0.3806	0.0012
M-VLDL-P	0.452	0.0001
M-VLDL-L	0.4464	0.0001
M-VLDL-PL	0.4574	0.0001
M-VLDL-C	0.4259	0.0002
M-VLDL-CE	0.353	0.0027
M-VLDL-FC	0.4623	0.0001
M-VLDL-TG	0.3935	0.0008
S-VLDL-P	0.4177	0.0003
S-VLDL-L	0.4265	0.0002
S-VLDL-PL	0.451	0.0001
S-VLDL-C	0.4363	0.0002
S-VLDL-CE	0.4226	0.0003
S-VLDL-FC	0.4516	0.0001
S-VLDL-TG	0.3646	0.0019
XS-VLDL-P	0.4245	0.0002
XS-VLDL-L	0.4244	0.0003
XS-VLDL-PL	0.4227	0.0003
XS-VLDL-C	0.392	0.0008
XS-VLDL-CE	0.3528	0.0027
XS-VLDL-FC	0.432	0.0002
XS-VLDL-TG	0.3681	0.0017
IDL-P	0.4013	0.0006
IDL-L	0.3492	0.003
IDL-PL	0.3159	0.0077
IDL-C	0.3115	0.0087
IDL-CE	0.3155	0.0078
IDL-FC	0.2921	0.0141
IDL-TG	0.3822	0.0011
L-LDL-P	0.4332	0.0002
L-LDL-L	0.3164	0.0076
L-LDL-PL	0.2986	0.012
L-LDL-C	0.2893	0.0151
L-LDL-CE	0.3037	0.0106
L-LDL-FC	0.2382	0.0471
L-LDL-TG	0.3885	0.0009
M-LDL-P	0.4094	0.0004
M-LDL-L	0.3931	0.0008
M-LDL-PL	0.3821	0.0011
M-LDL-C	0.3787	0.0012
M-LDL-CE	0.4015	0.0006
M-LDL-FC	0.275	0.0212
M-LDL-TG	0.3971	0.0007
S-LDL-P	0.4448	0.0001
S-LDL-L	0.439	0.0001
S-LDL-PL	0.4513	0.0001
S-LDL-C	0.4046	0.0005
S-LDL-CE	0.4187	0.0003
S-LDL-FC	0.3177	0.0074
S-LDL-TG	0.3849	0.001
XL-HDL-P	0.1378	0.2553
XL-HDL-L	0.05716	0.6383
XL-HDL-PL	0.02684	0.8255
XL-HDL-C	0.05191	0.6695
XL-HDL-CE	0.04363	0.7198
XL-HDL-FC	0.07937	0.5137
XL-HDL-TG	0.3402	0.004
L-HDL-P	-0.0778	0.5221
L-HDL-L	-0.1131	0.3511
L-HDL-PL	-0.138	0.2547
L-HDL-C	-0.1078	0.3746
L-HDL-CE	-0.1182	0.3299

L-HDL-FC	-0.06928	0.5687
L-HDL-TG	0.1585	0.19
M-HDL-P	-0.244	0.0418
M-HDL-L	-0.257	0.0317
M-HDL-PL	-0.2501	0.0368
M-HDL-C	-0.2876	0.0158
M-HDL-CE	-0.3071	0.0097
M-HDL-FC	-0.1861	0.1229
M-HDL-TG	0.1618	0.1807
S-HDL-P	-0.1172	0.3339
S-HDL-L	-0.1187	0.3277
S-HDL-PL	-0.1574	0.1933
S-HDL-C	-0.1636	0.1761
S-HDL-CE	-0.2216	0.0652
S-HDL-FC	0.09647	0.4269
S-HDL-TG	0.2736	0.0219
VLDL-D	0.2395	0.0458
LDL-D	0.1755	0.1461
HDL-D	-0.1003	0.4088
Serum-C	0.3322	0.005
VLDL-C	0.445	0.0001
Remnant-C	0.4427	0.0001
LDL-C	0.3305	0.0052
HDL-C	-0.1965	0.103
EstC	0.2936	0.0136
FreeC	0.4085	0.0004
Serum-TG	0.3664	0.0018
VLDL-TG	0.3667	0.0018
LDL-TG	0.3935	0.0008
HDL-TG	0.218	0.0699
TotPG	0.2032	0.0915
TG/PG	0.3666	0.0018
PC	0.2252	0.0608
SM	0.2806	0.0186
TotCho	0.2056	0.0878
ApoA1	0.4463	0.0001
ApoB	-0.1915	0.1123
ApoB/ApoA1	0.4579	0.0001
TotFA	0.3709	0.0016
UnSat	-0.2748	0.0213
DHA	0.06504	0.5927
LA	0.2417	0.0438
FAw3	0.146	0.2277
FAw6	0.2742	0.0216
PUFA	0.2695	0.0241
MUFA	0.3831	0.0011
SFA	0.3832	0.0011

Table S4: List of metabolites that significantly correlate with disease activity (SLEDAI) scores in JSLE: Key: brown (VLDL), green (HDL), grey (apolipoproteins), pink (fatty acids, %FA), yellow (other lipid related metabolites). P values from metabolites that were significantly correlated with SLEDAI following 10% FDR correction are shown in red.

	Flare		Pre-flare		Fold change	P value
	Mean	SD	Mean	SD		
XXL-VLDL-P	2.56E-10	2.07E-10	8.26E-11	2.58E-11	3.10E+00	1.57E-01
XXL-VLDL-L	5.49E-02	4.47E-02	1.73E-02	5.77E-03	3.17E+00	1.57E-01
XXL-VLDL-PL	6.32E-03	5.73E-03	1.63E-03	9.68E-04	3.88E+00	1.66E-01
XXL-VLDL-C	1.02E-02	8.72E-03	2.64E-03	1.43E-03	3.86E+00	1.40E-01
XXL-VLDL-CE	6.06E-03	4.87E-03	1.63E-03	7.79E-04	3.71E+00	1.18E-01
XXL-VLDL-FC	4.11E-03	3.89E-03	9.98E-04	7.11E-04	4.12E+00	1.73E-01
XXL-VLDL-TG	3.84E-02	3.03E-02	1.31E-02	3.45E-03	2.94E+00	1.61E-01
XL-VLDL-P	1.47E-09	1.31E-09	4.09E-10	2.75E-10	3.59E+00	1.60E-01
XL-VLDL-L	1.43E-01	1.28E-01	3.91E-02	2.67E-02	3.66E+00	1.59E-01
XL-VLDL-PL	2.27E-02	2.09E-02	4.96E-03	4.43E-03	4.58E+00	1.51E-01
XL-VLDL-C	2.93E-02	2.60E-02	7.38E-03	5.22E-03	3.97E+00	1.44E-01
XL-VLDL-CE	1.70E-02	1.45E-02	4.95E-03	2.96E-03	3.43E+00	1.39E-01
XL-VLDL-FC	1.24E-02	1.15E-02	2.43E-03	2.45E-03	5.08E+00	1.51E-01
XL-VLDL-TG	9.08E-02	8.07E-02	2.67E-02	1.72E-02	3.40E+00	1.66E-01
L-VLDL-P	9.24E-09	7.37E-09	3.16E-09	2.15E-09	2.92E+00	1.45E-01
L-VLDL-L	5.35E-01	4.28E-01	1.81E-01	1.24E-01	2.95E+00	1.44E-01
L-VLDL-PL	9.56E-02	7.71E-02	3.09E-02	2.22E-02	3.09E+00	1.43E-01
L-VLDL-C	1.25E-01	9.99E-02	4.07E-02	2.77E-02	3.06E+00	1.32E-01
L-VLDL-CE	6.92E-02	5.09E-02	2.55E-02	1.52E-02	2.71E+00	1.10E-01
L-VLDL-FC	5.55E-02	4.91E-02	1.53E-02	1.32E-02	3.64E+00	1.57E-01
L-VLDL-TG	3.15E-01	2.51E-01	1.09E-01	7.47E-02	2.87E+00	1.51E-01
M-VLDL-P	2.94E-08	1.88E-08	1.33E-08	6.12E-09	2.21E+00	1.38E-01
M-VLDL-L	9.78E-01	6.25E-01	4.40E-01	2.04E-01	2.22E+00	1.36E-01
M-VLDL-PL	1.92E-01	1.20E-01	8.72E-02	4.05E-02	2.20E+00	1.30E-01
M-VLDL-C	2.54E-01	1.54E-01	1.11E-01	5.33E-02	2.30E+00	1.05E-01
M-VLDL-CE	1.41E-01	7.54E-02	6.37E-02	2.73E-02	2.21E+00	7.80E-02
M-VLDL-FC	1.14E-01	7.95E-02	4.66E-02	2.68E-02	2.44E+00	1.41E-01
M-VLDL-TG	5.31E-01	3.53E-01	2.42E-01	1.13E-01	2.20E+00	1.54E-01
S-VLDL-P	4.14E-08	2.08E-08	2.30E-08	9.38E-09	1.80E+00	1.00E-01
S-VLDL-L	7.93E-01	3.90E-01	4.42E-01	1.86E-01	1.80E+00	9.37E-02
S-VLDL-PL	1.76E-01	8.34E-02	1.07E-01	4.25E-02	1.65E+00	8.79E-02
S-VLDL-C	2.43E-01	1.03E-01	1.33E-01	7.63E-02	1.82E+00	4.31E-02
S-VLDL-CE	1.39E-01	5.56E-02	7.53E-02	4.90E-02	1.85E+00	2.50E-02
S-VLDL-FC	1.04E-01	5.24E-02	5.81E-02	2.84E-02	1.79E+00	9.15E-02
S-VLDL-TG	3.74E-01	2.15E-01	2.02E-01	7.60E-02	1.85E+00	1.39E-01
XS-VLDL-P	3.94E-08	1.48E-08	2.69E-08	1.14E-08	1.46E+00	2.51E-02
XS-VLDL-L	4.82E-01	1.81E-01	3.34E-01	1.47E-01	1.44E+00	1.80E-02
XS-VLDL-PL	1.34E-01	5.23E-02	9.76E-02	4.61E-02	1.38E+00	5.27E-03
XS-VLDL-C	2.04E-01	8.45E-02	1.49E-01	7.72E-02	1.37E+00	5.51E-03
XS-VLDL-CE	1.36E-01	5.69E-02	9.94E-02	5.10E-02	1.36E+00	4.93E-03
XS-VLDL-FC	6.83E-02	2.84E-02	4.94E-02	2.70E-02	1.38E+00	1.46E-02
XS-VLDL-TG	1.44E-01	7.32E-02	8.78E-02	2.95E-02	1.64E+00	1.19E-01
IDL-P	9.17E-08	3.49E-08	7.17E-08	3.05E-08	1.28E+00	4.87E-03
IDL-L	9.09E-01	3.65E-01	7.16E-01	3.14E-01	1.27E+00	9.42E-03
IDL-PL	2.43E-01	9.84E-02	2.03E-01	8.03E-02	1.20E+00	4.38E-02
IDL-C	5.35E-01	2.52E-01	4.24E-01	2.12E-01	1.26E+00	3.48E-02
IDL-CE	3.91E-01	1.72E-01	3.00E-01	1.51E-01	1.30E+00	1.39E-02
IDL-FC	1.44E-01	8.12E-02	1.24E-01	6.10E-02	1.16E+00	2.68E-01
IDL-TG	1.30E-01	5.84E-02	8.98E-02	2.98E-02	1.45E+00	9.51E-02
L-LDL-P	1.50E-07	6.47E-08	1.10E-07	6.60E-08	1.36E+00	5.24E-03
L-LDL-L	1.05E+00	4.71E-01	7.84E-01	4.69E-01	1.34E+00	5.28E-03
L-LDL-PL	2.70E-01	9.74E-02	2.06E-01	1.16E-01	1.31E+00	2.16E-02
L-LDL-C	6.80E-01	3.57E-01	5.15E-01	3.12E-01	1.32E+00	1.84E-02
L-LDL-CE	4.91E-01	2.64E-01	3.61E-01	2.24E-01	1.36E+00	1.45E-02
L-LDL-FC	1.88E-01	9.45E-02	1.54E-01	8.89E-02	1.23E+00	4.87E-02
L-LDL-TG	1.03E-01	4.41E-02	6.29E-02	4.17E-02	1.64E+00	1.22E-01
M-LDL-P	1.16E-07	6.97E-08	8.98E-08	5.53E-08	1.29E+00	3.55E-02
M-LDL-L	5.88E-01	3.51E-01	4.59E-01	2.81E-01	1.28E+00	3.79E-02
M-LDL-PL	1.56E-01	8.91E-02	1.30E-01	7.42E-02	1.20E+00	3.31E-02
M-LDL-C	3.91E-01	2.35E-01	2.98E-01	1.86E-01	1.31E+00	5.17E-02
M-LDL-CE	2.83E-01	1.76E-01	2.05E-01	1.34E-01	1.38E+00	4.83E-02
M-LDL-FC	1.08E-01	6.01E-02	9.36E-02	5.29E-02	1.16E+00	7.15E-02
M-LDL-TG	4.09E-02	3.04E-02	3.13E-02	2.10E-02	1.31E+00	1.55E-01
S-LDL-P	1.35E-07	8.13E-08	1.07E-07	6.57E-08	1.26E+00	3.61E-02
S-LDL-L	3.78E-01	2.26E-01	3.02E-01	1.85E-01	1.25E+00	3.85E-02
S-LDL-PL	1.12E-01	6.32E-02	9.72E-02	5.56E-02	1.15E+00	3.26E-02
S-LDL-C	2.38E-01	1.45E-01	1.85E-01	1.17E-01	1.29E+00	5.48E-02
S-LDL-CE	1.73E-01	1.08E-01	1.28E-01	8.41E-02	1.35E+00	4.90E-02
S-LDL-FC	6.54E-02	3.71E-02	5.73E-02	3.35E-02	1.14E+00	9.88E-02
S-LDL-TG	2.77E-02	2.21E-02	1.98E-02	1.28E-02	1.40E+00	2.03E-01
XL-HDL-P	3.23E-07	1.53E-07	4.38E-07	1.30E-07	7.37E-01	2.31E-01
XL-HDL-L	3.21E-01	1.60E-01	4.41E-01	1.33E-01	7.29E-01	2.32E-01
XL-HDL-PL	1.46E-01	8.43E-02	2.24E-01	7.84E-02	6.52E-01	1.65E-01
XL-HDL-C	1.54E-01	9.17E-02	2.04E-01	6.28E-02	7.52E-01	3.01E-01
XL-HDL-CE	1.20E-01	6.84E-02	1.54E-01	4.47E-02	7.78E-01	3.26E-01
XL-HDL-FC	3.41E-02	2.39E-02	5.07E-02	1.90E-02	6.72E-01	2.55E-01
XL-HDL-TG	2.18E-02	1.04E-02	1.26E-02	1.76E-03	1.73E+00	1.52E-01
L-HDL-P	5.45E-07	4.79E-07	1.04E-06	2.97E-07	5.22E-01	7.12E-02

L-HDL-L	3.41E-01	3.02E-01	6.58E-01	1.92E-01	5.19E-01	6.82E-02
L-HDL-PL	1.65E-01	1.43E-01	3.04E-01	8.44E-02	5.43E-01	8.60E-02
L-HDL-C	1.60E-01	1.48E-01	3.27E-01	1.09E-01	4.90E-01	5.22E-02
L-HDL-CE	1.27E-01	1.16E-01	2.59E-01	8.25E-02	4.90E-01	5.27E-02
L-HDL-FC	3.35E-02	3.21E-02	6.87E-02	2.66E-02	4.88E-01	5.09E-02
L-HDL-TG	1.58E-02	1.30E-02	2.71E-02	4.96E-03	5.83E-01	2.11E-01
M-HDL-P	1.12E-06	6.64E-07	1.62E-06	3.65E-07	6.92E-01	1.48E-01
M-HDL-L	4.64E-01	2.85E-01	6.82E-01	1.60E-01	6.80E-01	1.45E-01
M-HDL-PL	2.24E-01	1.25E-01	3.24E-01	6.68E-02	6.92E-01	1.49E-01
M-HDL-C	2.03E-01	1.59E-01	3.22E-01	9.57E-02	6.32E-01	1.51E-01
M-HDL-CE	1.69E-01	1.31E-01	2.66E-01	7.66E-02	6.37E-01	1.41E-01
M-HDL-FC	3.41E-02	2.74E-02	5.62E-02	2.01E-02	6.07E-01	2.06E-01
M-HDL-TG	3.69E-02	2.34E-02	3.63E-02	9.14E-03	1.02E+00	9.50E-01
S-HDL-P	4.11E-06	5.48E-07	4.40E-06	7.21E-07	9.35E-01	4.16E-01
S-HDL-L	9.01E-01	1.34E-01	9.73E-01	1.63E-01	9.26E-01	3.81E-01
S-HDL-PL	4.75E-01	5.42E-02	5.28E-01	6.61E-02	9.01E-01	2.46E-01
S-HDL-C	3.55E-01	1.22E-01	3.96E-01	9.63E-02	8.97E-01	4.33E-01
S-HDL-CE	2.68E-01	1.15E-01	2.98E-01	8.16E-02	8.99E-01	5.03E-01
S-HDL-FC	8.67E-02	1.22E-02	9.75E-02	1.72E-02	8.90E-01	2.39E-01
S-HDL-TG	7.01E-02	2.94E-02	5.01E-02	1.30E-02	1.40E+00	1.20E-01
VLDL-D	3.79E+01	1.77E+00	3.70E+01	1.25E+00	1.02E+00	1.95E-01
LDL-D	2.36E+01	2.00E-01	2.36E+01	1.48E-01	1.00E+00	>0.999999
HDL-D	9.68E+00	3.03E-01	9.99E+00	2.06E-01	9.68E-01	1.44E-01
Serum-C	3.59E+00	1.24E+00	3.13E+00	1.12E+00	1.15E+00	5.38E-02
VLDL-C	8.66E-01	4.19E-01	4.44E-01	2.19E-01	1.95E+00	6.89E-02
Remnant-C	1.40E+00	5.09E-01	8.69E-01	4.06E-01	1.61E+00	2.87E-02
LDL-C	1.32E+00	7.22E-01	1.02E+00	5.89E-01	1.30E+00	3.71E-02
HDL-C	8.76E-01	4.36E-01	1.25E+00	2.27E-01	7.02E-01	1.07E-01
HDL2-C	4.56E-01	3.57E-01	7.80E-01	2.05E-01	5.85E-01	9.98E-02
HDL3-C	4.19E-01	1.19E-01	4.70E-01	3.62E-02	8.92E-01	3.38E-01
EstC	2.84E+00	9.09E-01	2.52E+00	5.41E-01	9.73E-01	1.63E-01
FreeC	1.25E+00	3.43E-01	1.06E+00	2.23E-01	1.08E+00	3.59E-02
Serum-TG	1.99E+00	1.18E+00	1.03E+00	3.68E-01	1.92E+00	1.41E-01
VLDL-TG	1.49E+00	9.99E-01	6.84E-01	2.99E-01	2.18E+00	1.51E-01
LDL-TG	1.91E-01	8.38E-02	1.34E-01	5.24E-02	1.43E+00	6.76E-02
HDL-TG	1.70E-01	5.87E-02	1.26E-01	2.05E-02	1.35E+00	1.23E-01
TotPG	1.63E+00	3.55E-01	1.50E+00	3.47E-01	1.03E+00	6.93E-02
TG/PG	8.30E-01	6.96E-01	6.28E-01	2.23E-01	1.80E+00	3.90E-01
PC	1.71E+00	3.48E-01	1.55E+00	3.34E-01	1.04E+00	9.84E-02
SM	3.77E-01	1.06E-01	3.63E-01	9.04E-02	9.32E-01	4.27E-01
TotCho	2.06E+00	3.96E-01	1.87E+00	3.72E-01	1.03E+00	9.02E-02
ApoA1	1.20E+00	2.16E-01	1.31E+00	1.88E-01	9.13E-01	1.88E-01
ApoB	9.22E-01	2.64E-01	6.43E-01	1.98E-01	1.43E+00	3.94E-02
ApoB/ApoA1	8.07E-01	2.86E-01	4.83E-01	1.15E-01	1.67E+00	8.14E-02
TotFA	1.13E+01	3.12E+00	9.37E+00	1.84E+00	1.21E+00	1.38E-01
UnSat	1.19E+00	1.13E-01	1.20E+00	7.28E-02	9.57E-01	7.87E-01
DHA	8.72E-02	1.48E-02	9.15E-02	2.14E-02	9.05E-01	7.56E-01
LA	3.19E+00	6.23E-01	2.84E+00	2.32E-01	1.03E+00	1.14E-01
FAw3	3.17E-01	4.68E-02	2.86E-01	7.45E-02	1.10E+00	5.34E-01
FAw6	3.67E+00	7.09E-01	3.32E+00	3.66E-01	1.02E+00	7.74E-02
PUFA	3.99E+00	7.38E-01	3.61E+00	4.20E-01	1.03E+00	1.02E-01
MUFA	3.46E+00	1.65E+00	2.46E+00	7.07E-01	1.50E+00	2.09E-01
SFA	3.86E+00	1.17E+00	3.29E+00	7.40E-01	1.21E+00	1.68E-01

Table S5: Metabolites significantly associated with a disease flare in JSLE: Key: brown (VLDL), orange (IDL), red (LDL) grey (apolipoproteins), yellow (other lipids), blue (glycolysis), black (other). P values from metabolites that were significantly correlated with lipid rafts following 10% FDR correction are shown in red.

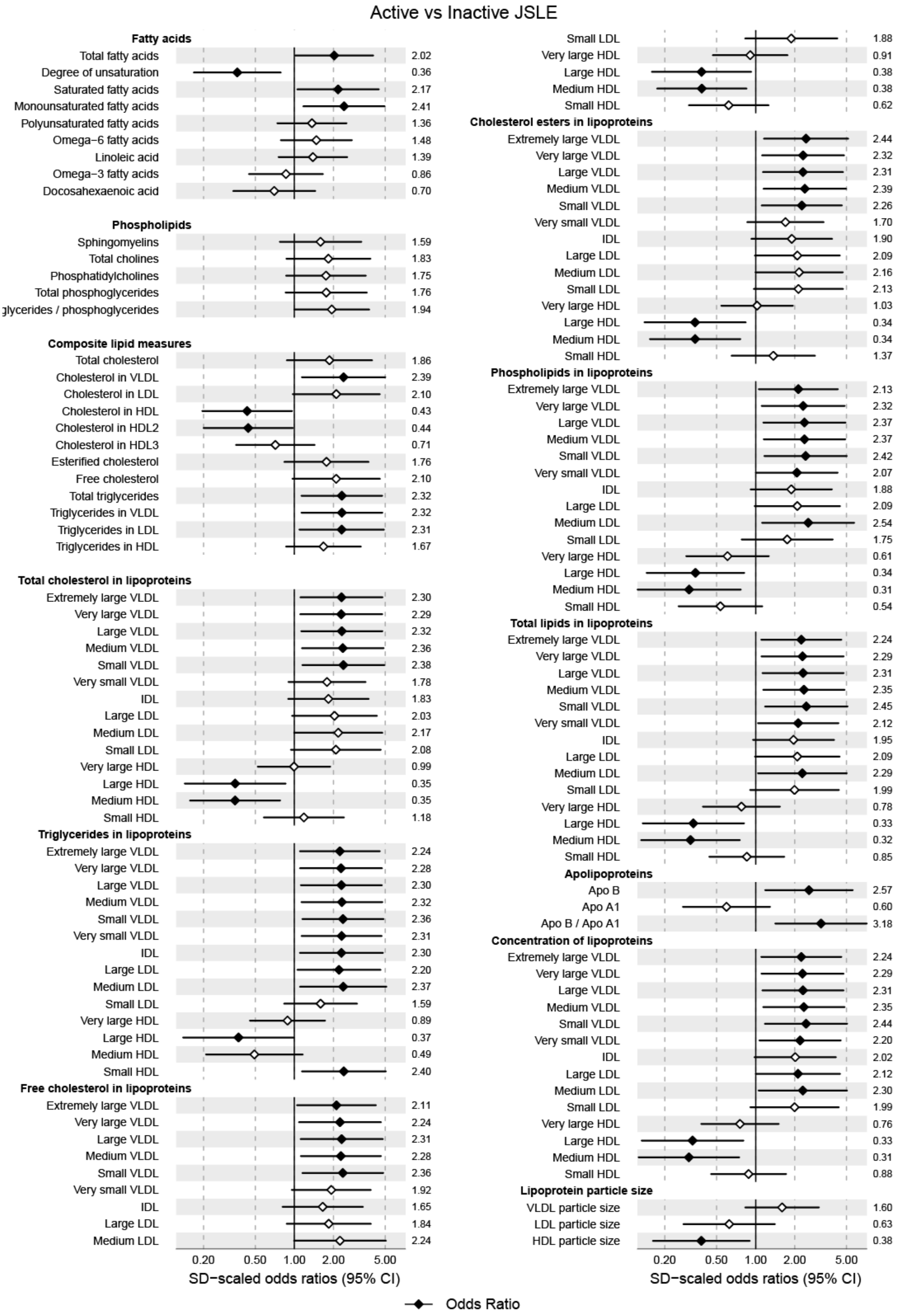

Figure S2: Atherogenic lipoproteins are associated with increased disease activity in JSLE. Forest plot showing OR and 95% CI of serum lipid metabolites between JSLE patients with active (n=22) vs inactive (n=43) disease by logistic regression analysis adjusted for age, sex, race and treatment. The concentration of total lipids (mmol/l), apolipoproteins (g/l) and

lipoprotein measurements including particle size (nm), concentration and lipid content (mmol/l) are displayed. Statistically significant differences denoted by solid black diamond; non-statistically significant differences denoted by open diamond. Abbreviations: Apo, apolipoprotein; VLDL, very low density lipoprotein; IDL, intermediate density lipoprotein; LDL, low density lipoprotein; HDL, high density lipoprotein.

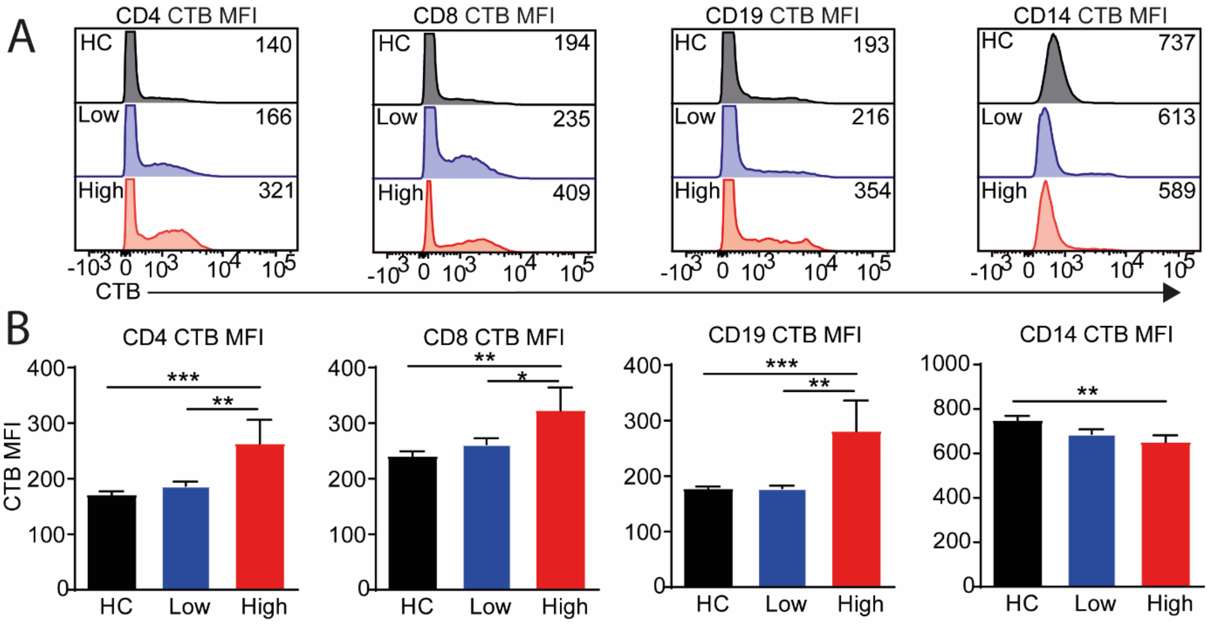

Figure S3: Immune cells lipid raft expression in JSLE patients. A) Representative histograms and **(B)** bar graphs displaying CTB expression (MFI) values of CD4, 8 T-cell, CD19 B-cell and CD14 monocyte in high activity (SLEDAI≥4, n=15, red) low activity (SLEDAI<4, n=20, blue) JSLE patients and healthy controls (n=39) (Black). PBMCs were stained with fluorochrome-labelled antibodies (CD4, CD8, CD19 & CD14) as well as CTB to detect glycosphingolipid expression in immune cell subsets. Samples were analysed using Fortessa X20 flow cytometer and Flowjo software. Mean, t-test (*=P<0.05, **=P<0.01, ***=P<0.001, ****=P<0.0001.

	CD4		CD8		CD19		CD14	
**Metabolite**	**R** (pearson)	**P**	**R** (pearson)	**P**	**R** (pearson)	**P**	**R** (pearson)	**P**
XXL-VLDL-P	0.1771	0.3242	0.215	0.2294	0.1406	0.4429	-0.07913	0.6616
XXL-VLDL-L	0.1803	0.3155	0.2184	0.2221	0.1413	0.4406	-0.07786	0.6667
XXL-VLDL-PL	0.1461	0.4173	0.1864	0.2989	0.08682	0.6366	-0.06092	0.7363
XXL-VLDL-C	0.239	0.1804	0.2737	0.1232	0.2004	0.2715	-0.07669	0.6714
XXL-VLDL-CE	0.2811	0.113	0.31	0.0791	0.2729	0.1308	-0.09941	0.582
XXL-VLDL-FC	0.1808	0.3139	0.2228	0.2127	0.1039	0.5714	-0.04747	0.7931
XXL-VLDL-TG	0.1683	0.3492	0.2067	0.2483	0.1347	0.4625	-0.08074	0.6551
XL-VLDL-P	0.1941	0.2791	0.2446	0.1701	0.1292	0.4811	-0.05941	0.7426
XL-VLDL-L	0.1959	0.2747	0.2457	0.1682	0.1319	0.4716	-0.06038	0.7385
XL-VLDL-PL	0.1958	0.2748	0.241	0.1767	0.131	0.4749	-0.05499	0.7612
XL-VLDL-C	0.2187	0.2214	0.2614	0.1417	0.1709	0.3497	-0.07193	0.6908
XL-VLDL-CE	0.2322	0.1936	0.2751	0.1213	0.1977	0.2781	-0.08557	0.6359
XL-VLDL-FC	0.2003	0.2638	0.2425	0.1738	0.136	0.4581	-0.05406	0.7651
XL-VLDL-TG	0.1884	0.2937	0.2413	0.1762	0.12	0.5131	-0.05784	0.7492
L-VLDL-P	0.2171	0.2249	0.2714	0.1265	0.1734	0.3427	-0.05443	0.7635
L-VLDL-L	0.2182	0.2225	0.272	0.1257	0.1747	0.3389	-0.05432	0.764
L-VLDL-PL	0.2268	0.2044	0.2789	0.116	0.186	0.308	-0.05167	0.7752
L-VLDL-C	0.2317	0.1945	0.2802	0.1143	0.1985	0.2762	-0.0613	0.7347
L-VLDL-CE	0.2532	0.1552	0.2997	0.0902	0.2445	0.1775	-0.07116	0.6939
L-VLDL-FC	0.2063	0.2494	0.2565	0.1496	0.1456	0.4265	-0.05115	0.7774
L-VLDL-TG	0.2104	0.2399	0.2666	0.1337	0.1617	0.3766	-0.05273	0.7707
M-VLDL-P	0.237	0.1842	0.2895	0.1022	0.2216	0.2228	-0.04505	0.8034
M-VLDL-L	0.2409	0.1769	0.2925	0.0986	0.2274	0.2108	-0.04534	0.8022
M-VLDL-PL	0.2478	0.1644	0.2985	0.0915	0.2393	0.1871	-0.04092	0.8211
M-VLDL-C	0.2861	0.1064	0.3273	0.063	0.2955	0.1005	-0.04687	0.7956
M-VLDL-CE	0.324	0.0658	0.3556	0.0422	0.3554	0.0459	-0.05451	0.7632
M-VLDL-FC	0.2448	0.1698	0.2949	0.0957	0.227	0.2116	-0.03872	0.8306
M-VLDL-TG	0.2166	0.226	0.273	0.1243	0.1891	0.2998	-0.04527	0.8025
S-VLDL-P	0.2945	0.0961	0.3323	0.0588	0.304	0.0907	-0.02193	0.9036
S-VLDL-L	0.3008	0.089	0.3364	0.0556	0.3148	0.0793	-0.02246	0.9012
S-VLDL-PL	0.2848	0.1081	0.3183	0.071	0.2845	0.1146	-0.0163	0.9283
S-VLDL-C	0.3587	0.0404	0.3741	0.032	0.4176	0.0174	-0.02188	0.9038
S-VLDL-CE	0.3832	0.0277	0.384	0.0274	0.4694	0.0067	-0.0225	0.9011
S-VLDL-FC	0.2969	0.0933	0.3302	0.0606	0.3096	0.0847	-0.01773	0.922
S-VLDL-TG	0.2617	0.1413	0.3081	0.0811	0.2455	0.1756	-0.0226	0.9007
XS-VLDL-P	0.4191	0.0152	0.4084	0.0183	0.4961	0.0039	0.02107	0.9074
XS-VLDL-L	0.4177	0.0156	0.4017	0.0205	0.5055	0.0032	0.02144	0.9057
XS-VLDL-PL	0.3855	0.0267	0.3544	0.043	0.4984	0.0037	0.001961	0.9914
XS-VLDL-C	0.4059	0.0191	0.3636	0.0375	0.5527	0.001	0.04279	0.8131
XS-VLDL-CE	0.4229	0.0142	0.3705	0.0338	0.582	0.0005	0.07365	0.6838
XS-VLDL-FC	0.3422	0.0513	0.3235	0.0663	0.4604	0.008	-0.02562	0.8874
XS-VLDL-TG	0.331	0.0599	0.3624	0.0382	0.3451	0.053	0.004226	0.9814
VLDL-D	0.1402	0.4365	0.2153	0.2289	0.1059	0.5642	0.06889	0.7032
VLDL-C	0.3411	0.0521	0.3654	0.0365	0.3844	0.0298	-0.03259	0.8571
VLDL-TG	0.2264	0.2052	0.2795	0.1152	0.1992	0.2744	-0.04018	0.8243
RemNAnt-C	0.3732	0.0324	0.3723	0.0329	0.4548	0.0089	-0.013	0.9428
IDL-P	0.3688	0.0347	0.3219	0.0678	0.5321	0.0017	0.04489	0.8041
IDL-L	0.3504	0.0456	0.3002	0.0896	0.5301	0.0018	0.04246	0.8145
IDL-PL	0.3177	0.0716	0.2602	0.1436	0.5306	0.0018	0.046	0.7994
IDL-C	0.2929	0.0981	0.2419	0.175	0.5067	0.0031	0.02926	0.8716
IDL-CE	0.3085	0.0807	0.2643	0.1373	0.5035	0.0033	0.03098	0.8641
IDL-FC	0.2438	0.1716	0.1769	0.3247	0.5005	0.0035	0.0231	0.8985
IDL-TG	0.4399	0.0104	0.434	0.0116	0.4561	0.0087	0.06572	0.7163
L-LDL-P	0.2997	0.0902	0.2586	0.1462	0.4893	0.0045	0.02631	0.8845
L-LDL-L	0.2819	0.112	0.2397	0.1792	0.4825	0.0052	0.02057	0.9095
L-LDL-PL	0.2662	0.1342	0.2241	0.21	0.474	0.0061	0.0188	0.9173
L-LDL-C	0.2488	0.1626	0.2069	0.2479	0.468	0.0069	0.01071	0.9528
L-LDL-CE	0.2531	0.1553	0.217	0.2252	0.4595	0.0081	0.005423	0.9761
L-LDL-FC	0.2256	0.2068	0.1672	0.3523	0.4871	0.0047	0.02791	0.8775
L-LDL-TG	0.4643	0.0065	0.4349	0.0114	0.4814	0.0053	0.1059	0.5577
M-LDL-P	0.2598	0.1442	0.2269	0.2042	0.4557	0.0088	0.01331	0.9414
M-LDL-L	0.2469	0.1661	0.2136	0.2327	0.4513	0.0095	0.01235	0.9456
M-LDL-PL	0.2628	0.1395	0.2398	0.1789	0.4354	0.0128	0.01906	0.9162
M-LDL-C	0.2119	0.2364	0.1776	0.3229	0.4374	0.0123	0.001312	0.9942
M-LDL-CE	0.2161	0.2271	0.1827	0.3089	0.4354	0.0127	-0.004181	0.9816
M-LDL-FC	0.1801	0.3159	0.1435	0.4256	0.4316	0.0136	0.02137	0.906
M-LDL-TG	0.4298	0.0125	0.3967	0.0223	0.4455	0.0106	0.09694	0.5915
S-LDL-P	0.135	0.4537	0.113	0.5312	0.3958	0.025	-0.003242	0.9857
S-LDL-L	0.13	0.4709	0.1069	0.5537	0.3923	0.0264	-0.004943	0.9782
S-LDL-PL	0.02416	0.8938	0.01903	0.9163	0.3102	0.084	-0.009251	0.9593
S-LDL-C	0.1573	0.3821	0.1258	0.4854	0.4056	0.0213	-0.01197	0.9473
S-LDL-CE	0.1884	0.2936	0.1523	0.3976	0.4186	0.0171	-0.0115	0.9493
S-LDL-FC	0.04525	0.8026	0.0309	0.8645	0.3353	0.0606	-0.01341	0.941
S-LDL-TG	0.1814	0.3124	0.1812	0.313	0.3624	0.0415	0.05779	0.7494
LDL-D	0.2717	0.1262	0.2263	0.2053	0.09469	0.6062	0.02403	0.8944

LDL-C	0.2205	0.2175	0.1827	0.3088	0.4467	0.0104	0.001967	0.9913
LDL-TG	0.4318	0.0121	0.4085	0.0183	0.4469	0.0103	0.08453	0.64
XL-HDL-P	-0.2231	0.212	-0.3104	0.0787	-0.1977	0.278	-0.1418	0.4311
XL-HDL-L	-0.2155	0.2284	-0.3022	0.0874	-0.1887	0.301	-0.1374	0.4458
XL-HDL-PL	-0.2535	0.1546	-0.3407	0.0523	-0.2602	0.1504	-0.1531	0.395
XL-HDL-C	-0.1537	0.393	-0.2328	0.1924	-0.09746	0.5957	-0.1072	0.5527
XL-HDL-CE	-0.1593	0.3759	-0.2365	0.1851	-0.09525	0.6041	-0.1081	0.5494
XL-HDL-FC	-0.1355	0.4522	-0.2157	0.2281	-0.09952	0.5879	-0.09971	0.5809
XL-HDL-TG	-0.1434	0.426	-0.1959	0.2746	-0.04463	0.8084	-0.1007	0.5771
L-HDL-P	-0.3157	0.0735	-0.3772	0.0305	-0.3985	0.0239	-0.1019	0.5727
L-HDL-L	-0.3197	0.0697	-0.3811	0.0287	-0.4028	0.0223	-0.1082	0.5488
L-HDL-PL	-0.3271	0.0631	-0.3875	0.0259	-0.4161	0.0179	-0.1028	0.5691
L-HDL-C	-0.3331	0.0582	-0.392	0.024	-0.4113	0.0193	-0.1244	0.4905
L-HDL-CE	-0.3317	0.0594	-0.3899	0.0249	-0.4107	0.0195	-0.1157	0.5213
L-HDL-FC	-0.3378	0.0545	-0.3976	0.0219	-0.4116	0.0192	-0.1538	0.3928
L-HDL-TG	0.04411	0.8074	-0.02679	0.8823	0.02	0.9135	0.08182	0.6508
M-HDL-P	-0.3547	0.0429	-0.3629	0.038	-0.446	0.0105*	0.03664	0.8396
M-HDL-L	-0.3653	0.0366	-0.3729	0.0326	-0.46	0.0081	0.02675	0.8825
M-HDL-PL	-0.3205	0.069	-0.3379	0.0544	-0.4051	0.0214	0.06172	0.733
M-HDL-C	-0.4376	0.0109	-0.4364	0.0111	-0.5458	0.0012	-0.03148	0.8619
M-HDL-CE	-0.4434	0.0098	-0.4404	0.0103	-0.5519	0.0011	-0.03145	0.8621
M-HDL-FC	-0.4075	0.0186	-0.4152	0.0163	-0.5119	0.0027	-0.03038	0.8667
M-HDL-TG	0.1051	0.5605	0.09812	0.587	0.1406	0.4428	0.2771	0.1185
S-HDL-P	-0.2942	0.0965	-0.2592	0.1452	-0.297	0.0988	0.1167	0.5178
S-HDL-L	-0.3103	0.0789	-0.279	0.1158	-0.3136	0.0805	0.1111	0.5382
S-HDL-PL	-0.3386	0.0539	-0.2908	0.1007	-0.4202	0.0166	0.07954	0.6599
S-HDL-C	-0.2254	0.2073	-0.2386	0.1812	-0.08484	0.6443	0.07198	0.6906
S-HDL-CE	-0.1542	0.3916	-0.1788	0.3195	0.04662	0.8	0.05571	0.7581
S-HDL-FC	-0.3679	0.0352	-0.3244	0.0655	-0.4186	0.0171	0.09238	0.6091
S-HDL-TG	0.3257	0.0644	0.3603	0.0394	0.3176	0.0765	0.07049	0.6967
HDL-D	-0.303	0.0865	-0.3815	0.0285	-0.3358	0.0603	-0.1172	0.516
HDL-C	-0.4025	0.0202	-0.4545	0.0079	-0.4713	0.0065	-0.08973	0.6195
HDL2-C	-0.4031	0.02	-0.4487	0.0088	-0.4771	0.0058	-0.1127	0.5323
HDL3-C	-0.2153	0.2289	-0.2771	0.1185	-0.0182	0.9213	0.07572	0.6754
HDL-TG	0.3108	0.0784	0.3185	0.0708	0.2411	0.1838	0.01576	0.9306
Serum-C	0.1739	0.3331	0.1205	0.5041	0.3821	0.0309	-0.05143	0.7762
EstC	0.1229	0.4956	0.07217	0.6898	0.3493	0.0501	-0.05494	0.7614
FreeC	0.2827	0.1109	0.2274	0.2032	0.4394	0.0119	-0.04432	0.8065
Serum-TG	0.2624	0.1401	0.3058	0.0835	0.2445	0.1775	-0.02381	0.8953
TotPG	0.02179	0.9042	-0.01308	0.9424	0.007563	0.9672	-0.1422	0.4299
PC	0.04948	0.7845	0.01028	0.9547	0.04752	0.7962	-0.1615	0.3693
SM	0.06542	0.7176	-0.03402	0.8509	0.345	0.0531	-0.007993	0.9648
TotCho	0.04437	0.8063	-0.01141	0.9498	0.07933	0.666	-0.1602	0.3731
ApoA1	-0.3932	0.0236	-0.4408	0.0102	-0.4468	0.0104	-0.1332	0.4598
ApoB	0.3268	0.0634	0.3374	0.0549	0.4077	0.0206	-0.02905	0.8725
ApoB/ApoA1	0.4065	0.0189	0.4337	0.0117	0.4808	0.0053	-0.005907	0.974
TotFA	0.1989	0.267	0.2127	0.2346	0.2204	0.2256	-0.05965	0.7416
UnSat	-0.2776	0.1178	-0.3411	0.052	-0.2294	0.2066	-0.01216	0.9465
DHA	-0.1594	0.3757	-0.2414	0.1759	-0.1568	0.3915	0.03075	0.8651
LA	0.08151	0.652	0.07436	0.6809	0.2438	0.1787	-0.0403	0.8238
FAw3	0.006322	0.9721	-0.04747	0.7931	-0.01154	0.95	0.03154	0.8617
FAw6	0.08366	0.6435	0.06769	0.7082	0.2398	0.1861	-0.04829	0.7896
PUFA	0.07807	0.6659	0.05662	0.7543	0.2164	0.2342	-0.03776	0.8347
MUFA	0.2233	0.2115	0.251	0.1589	0.2282	0.2091	-0.04382	0.8087
SFA	0.212	0.2363	0.228	0.202	0.192	0.2926	-0.08292	0.6464

Table S6: Pearson correlation coefficients and p values from the correlation between JSLE patient lipid raft expression on immune cell subsets and serum lipids (metabolomics): Significant P values (p>0.05) corrected for multiple testing (10% FDR) are displayed in red. Abbreviations: TG (triglycerides), Est (esterified), PG (Phosphoglyceride), PC (Phosphatidylcholine), SM (Sphingomyelins), Unsat (Unsaturated), LA (Linoleic acid), FAw3 (Omega-3 fatty acids), FAw6 (Omega-6 fatty acids), PUFA (Polyunsaturated fatty acids), MUFA (Monounsaturated fatty acids), SFA (Saturated fatty acids), VLDL, IDL, LDL, HDL (very low, intermediate, low, high density lipoprotein), P (particles), C (cholesterol), CE (cholesterol esters), FC (free cholesterol).

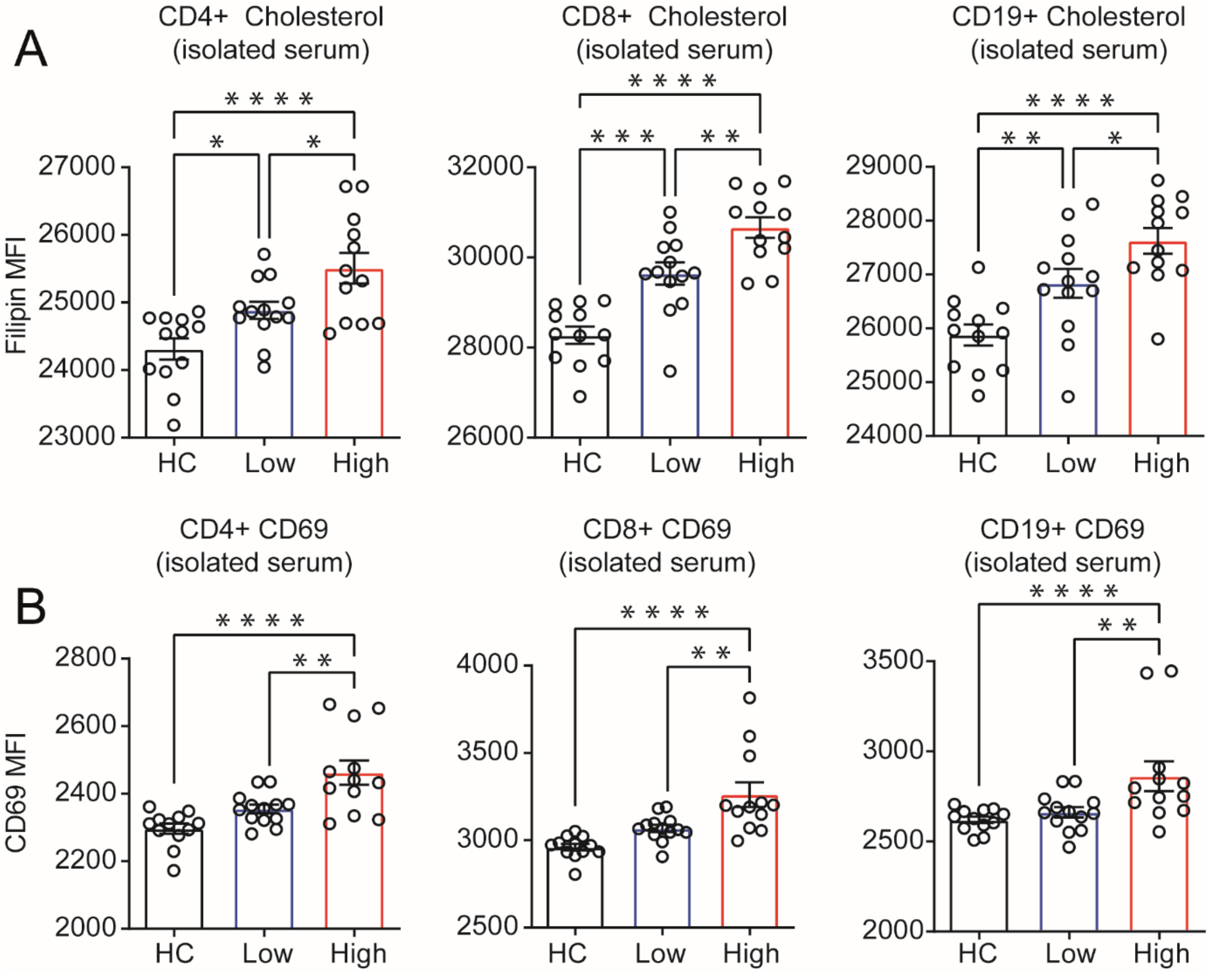

Figure S4: Immune cell lipid metabolism is stimulated by in vitro culture with serum from JSLE patients with active disease. Dot plots displaying the expression (MFI, flow cytometry) of **A)** filipin (cholesterol) or **B)** CD69 (activation) in HC lymphocytes following *in vitro* culture of PBMCs with individual serum isolated individual active (n=13) and inactive (n=13) JSLE patients and HCs (n=12). PBMCs were cultured for 48hrs in RPMI supplemented with 10% donor serum. Unpaired t test. Mean. SEM. *=P<0.05, **=P<0.01, ***=P<0.001, ****=P<0.0001.

1. Croca, S., et al., *IgG anti-apolipoprotein A-1 antibodies in patients with systemic lupus erythematosus are associated with disease activity and corticosteroid therapy: an observational study.* Arthritis Research & Therapy, 2015. **17**.
